# Corticotropin-Releasing Factor Family: A Stress Hormone-Receptor System’s Emerging Role in Mediating Sex-Specific Signaling

**DOI:** 10.3390/cells9040839

**Published:** 2020-03-31

**Authors:** Lahari Vuppaladhadiam, Cameron Ehsan, Meghana Akkati, Aditi Bhargava

**Affiliations:** 1 Department of Obstetrics, Gynecology & Reproductive Sciences, University of California, San Francisco, 513 Parnassus Avenue, San Francisco, CA 94143-0556, USA; lahari.vuppala2018@gmail.com (L.V.); cameronehsan@gmail.com (C.E.); meghanaakkati@gmail.com (M.A.); 2 The Osher Center for Integrative Medicine, University of California, San Francisco, 513 Parnassus Avenue, San Francisco, CA 94143-0556, USA

**Keywords:** BNST, cardiovascular, CRF, diabetes, GPCR, gut, inflammatory bowel disease, pancreas, reproduction, sexually dimorphic, urocortins

## Abstract

No organ in the body is impervious to the effects of stress, and a coordinated response from all organs is essential to deal with stressors. A dysregulated stress response that fails to bring systems back to homeostasis leads to compromised function and ultimately a diseased state. The components of the corticotropin-releasing factor (CRF) family, an ancient and evolutionarily conserved stress hormone-receptor system, helps both initiate stress responses and bring systems back to homeostasis once the stressors are removed. The mammalian CRF family comprises of four known agonists, CRF and urocortins (UCN1–3), and two known G protein-coupled receptors (GPCRs), CRF_1_ and CRF_2_. Evolutionarily, precursors of CRF- and urocortin-like peptides and their receptors were involved in osmoregulation/diuretic functions, in addition to nutrient sensing. Both CRF and UCN1 peptide hormones as well as their receptors appeared after a duplication event nearly 400 million years ago. All four agonists and both CRF receptors show sex-specific changes in expression and/or function, and single nucleotide polymorphisms are associated with a plethora of human diseases. CRF receptors harbor N-terminal cleavable peptide sequences, conferring biased ligand properties. CRF receptors have the ability to heteromerize with each other as well as with other GPCRs. Taken together, CRF receptors and their agonists due to their versatile functional adaptability mediate nuanced responses and are uniquely positioned to orchestrate sex-specific signaling and function in several tissues.

## 1. Introduction: The Corticotropin-Releasing Factor and Receptor Family

**Figure 1:**
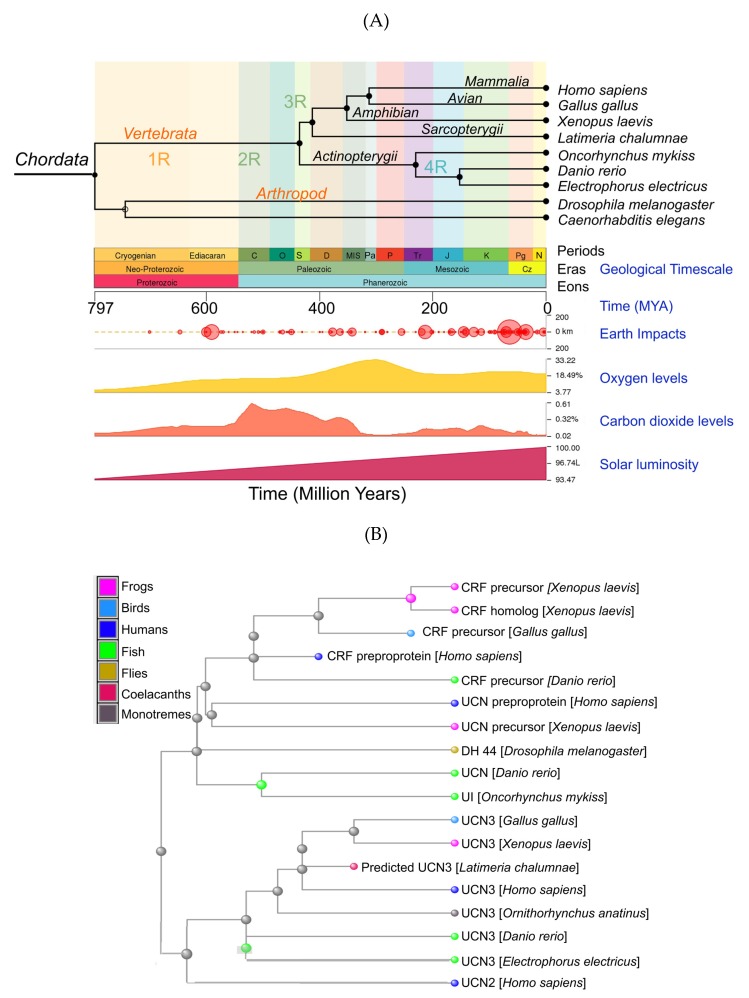

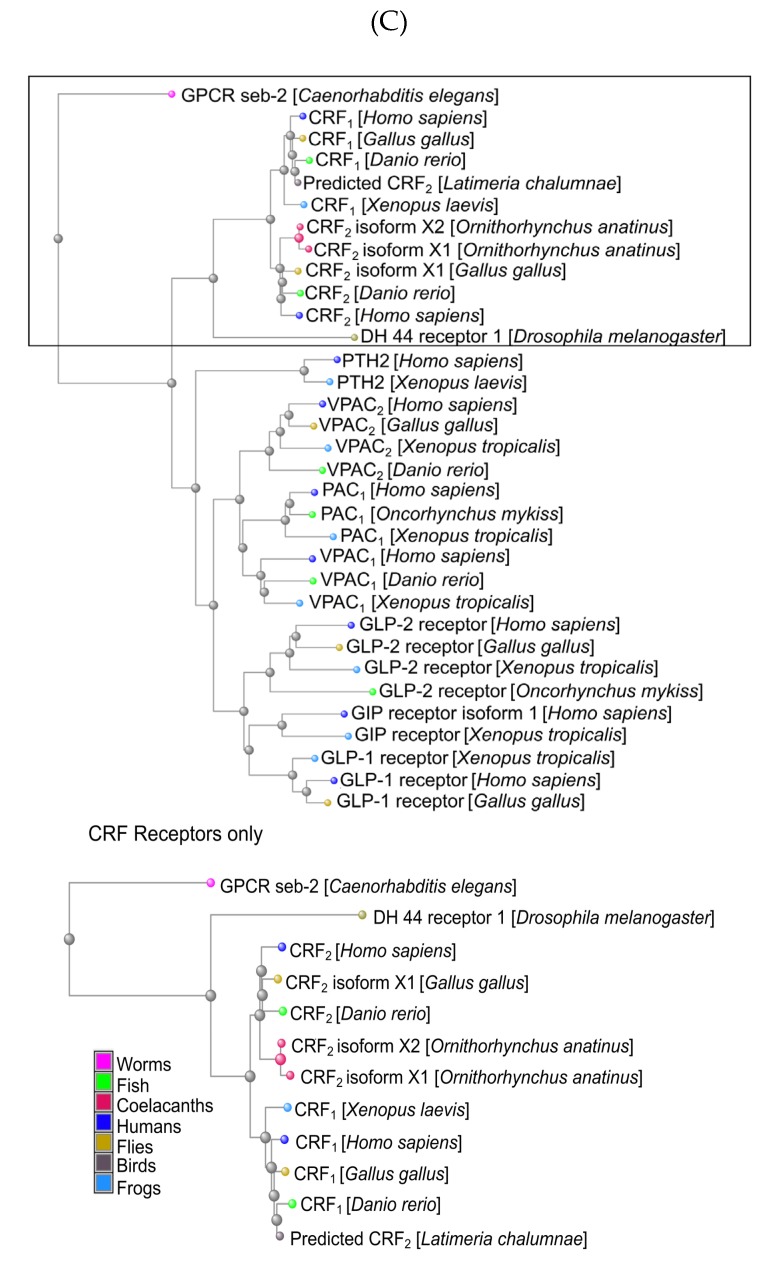
Origin of the corticotropin-releasing factor (CRF) family of peptide hormones: (**A**) Phylogenetic tree of showing evolution of prototype urotensin-I (UI), urocortins, and CRF with respect to geological times (in million years). The phylogenetic tree was generated using the TimeTree, which is a public knowledge base as described by Kumar et al. [4]. Prototype diuretic peptide hormones are present in the fruit fly, *Drosophila melanogaster*. Putative rounds of whole genome duplication events are depicted as 1R–4R. Earth impact events, changes in oxygen and carbon dioxide levels, as well as solar luminosity that might have influenced evolution are also shown. (**B**) A cluster dendogram of the CRF family of peptides was generated in Cobalt using the National Center for Biotechnology Information (NCBI) database. These data suggest that UCN3 and UCN2 evolved earlier than UCN1 and CRF, with UCN3 and UCN2 being more homologous to each other than to CRF and UCN1. UCN3 appears to be most conserved evolutionarily. (**C**) Cluster dendograms of CRF receptors and their orthologs. Boxed Inset: CRF receptors are more closely related to diuretic hormone 44 (DH44) receptors found in flies and arose from a common GPCR ancestral protein, seb-2, which is present in worms. CRF receptor orthologs: GIP: gastric inhibitory polypeptide receptor; GLP-1/2 R: glucagon-like peptide 1/2 receptors; PTH2: parathyroid hormone 2 receptor; PAC_1_: pituitary adenylate cyclase-activating polypeptide type I receptors; VPAC_1/2_; vasoactive intestinal polypeptide receptors 1 and 2.

Corticotropin-releasing factor/hormone (CRF or CRH), urocortins, their paralogs, urotensin-I and sauvagine, and their two known G protein-coupled receptors, CRF_1_ and CRF_2_, are a family of ancient peptide hormones found in all chordates (Figure 1A–C). A diuretic hormone found in the fruit fly, *Drosophila melanogaster*, shows homology with the mammalian CRF and urocortins and likely served as a potential peptide precursor in early metazoan ancestry nearly 800 million years ago (Figure 1A,B). The mammalian CRF receptors and the fly diuretic hormone receptors appear to have evolved from a common ancestral G protein-coupled receptor, seb-2, which is present in worms, including *Caenorhabditis elegans* (Figure 1C). Phylogenetically, urotensin-I appears to have evolved first and is found in brain regions that evolved earlier and regulated physiological functions such as osmo- and vasoregulation [1,2]; these functions are still shared by all homologs of the urotensin family. CRF is phylogenetically a younger peptide that evolved after duplication events [1,2] and is more prevalent in younger and higher order brain areas. CRF has overlapping physiological functions with the three urocortins, and together, these peptides regulate glucocorticoid and catecholamine release, energy metabolism, reproduction, cardiovascular, and immune functions, to list a few [3].

**Figure 2:**
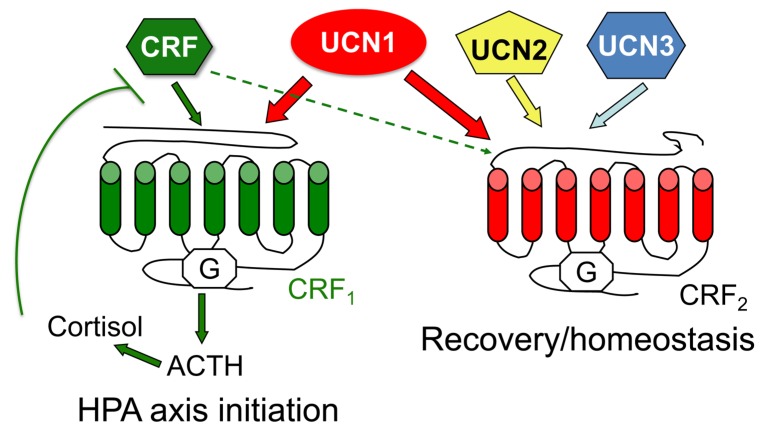
CRF family of peptide hormones and their two G protein-coupled receptors (GPCR) receptors, CRF_1_ and CRF_1_. Based on in vitro binding assays, CRF binds mainly to CRF_1_, whereas urocortin 1 (UCN1) binds to both receptors, but with 10-fold higher affinity than other ligands. UCN2 and UCN3 bind exclusively to CRF_2_. The activation of CRF_1_ receptors by CRF in the hypothalamus initiates the hypothalamic–pituitary–adrenal (HPA) axis, ultimately resulting in the release of glucocorticoids (cortisol in primates and corticosterone in rodents) from the adrenal cortex. Activation of CRF_2_ by urocortins is key for recovery from stress responses and returning to homeostasis.

The mammalian CRF family comprises of four known agonists, CRF and three urocortins (UCN1–3), and two known G protein-coupled receptors (GPCRs), CRF_1_ and CRF_2_ (Figure 2). In mammals, CRF is primarily responsible for regulating and/or initiating stress responses via activation of the hypothalamic–pituitary–adrenal (HPA) axis [5], whereas urocortins are involved in the recovery response to stress [6]. In peripheral organs, components of the CRF system are synthesized locally as in vivo RNA interference (RNAi) silenced its expression and altered gut function [7,8,9]. CRF and UCNs exhibit both autocrine and paracrine actions. While the actions of the CRF system in several processes are extensively researched as summarized in excellent reviews and references therein [3,4], the sex-specific modalities of the system are poorly understood. Female animals are generally not used in experiments, which led to the understanding of the role of the CRF system based on data primarily obtained from experiments based on male animals. Given that a myriad of diseases and disorders affecting tissues and organs in which the CRF system is expressed show sex-specific manifestation of diseases and outcomes, the need to characterize and elucidate the action and function of the CRF system in both sexes is needed. For example, epidemiological data suggest that the incidence of mental health and functional gastrointestinal disorders is higher in women than men [10,11], whereas rates of heart disease are higher in men than women [12,13], but how the actions of the CRF system differs between males and females in these organs is poorly understood. In this review, we will first touch upon the components of the CRF system and next review literature about what is known about the sex-specific actions of these hormones and their receptors.

## 2. The Hypothalamic–Pituitary–Adrenal (HPA) Axis

In the brain, CRF is synthesized by the paraventricular nucleus (PVN) and stored in the median eminence region of the hypothalamus [3]. In response to stress, CRF is released into the portal circulation from the median eminence, which in turn stimulates the secretion of adrenocorticotrophic hormone (ACTH) from the anterior pituitary into the bloodstream. ACTH goes on to regulate the release of glucocorticoids (cortisol in humans and corticosteroids in rodents) from the adrenal cortex. While urocortins are not known to directly activate the HPA axis, UCN1 activates the HPA axis by stimulating CRF production [14]. Elevated levels of cortisol during the stress response result in an increased concentration of amino acids in the blood, the increased release of fatty acids, and increased blood glucose levels as a result of stimulating the breakdown of non-carbohydrate sources. Elevated glucocorticoid levels as seen during chronic stress can have several adverse effects on physiology that are well-documented [15]. HPA axis function is also implicated in biology of addiction and alcohol dependence [16]. While genomic and non-genomic actions of glucocorticoids [17,18] that result in increased intracellular levels of Ca^2+^ are also well known, the mechanisms behind this increased Ca^2+^ levels in neurons that are less studied include extrusion or clearance of toxic levels of intracellular Ca^2+^, which can ultimately lead to neuronal cell death [19,20]. Moreover, hormones of the hypothalamic–pituitary–adrenal and gonadal axes regulate a plethora of functions that include immune, metabolic, and reproduction [21,22].

## 3. Looking Beyond the Fight or Flight Stress Response

The body’s integration of the nervous and endocrine systems in response to stress is critical to maintaining homeostasis, a term coined by Walter Cannon in 1929 [23]. The evolutionarily conserved survival mechanism that coordinates the reactions to stress in vertebrates was termed as the “fight or flight” response by Hans Selye [24,25], but coordination of stress responses requires orchestration of several organs and their functions [15]. The hypothalamus simultaneously activates the adrenal cortex via the HPA axis to release glucocorticoids and the sympathetic nervous system, which in turn secretes the hormones epinephrine and norepinephrine. Together, these circulating hormones in the bloodstream lead to many physiological changes such as elevated heartbeat, heart rate, and blood pressure; an increase in blood glucose levels; dilation of air passages; vasodilation near skeletal muscles and vasoconstriction around digestive organs; and dilation of pupils [26]. This response is referred to as the short-term alarm stage. The endocrine system, on the other hand, is responsible for sustaining the stress response in the long-term resistance stage.

A body of literature suggests that the components of the CRF system regulate several more biological processes other than activation of the HPA axis. For example, in rodents, CRF modulates feeding behavior under conditions of stress [27,28,29]. The injection of UCN1-3 in rodent brains decreases food intake after 90 min (in food-deprived animals) or 4–5 h (in *ad lib* fed). In these studies, effects were transient and seen at a very high dose [30,31]. All of these studies used only male rats; thus, it is not clear if UCNs have any long-lasting effect on food intake, or in female animals. UCN2 infusion over days also modulated food intake in male mice [32,33]. Thus, nutrient sensing appears to be an ancient function that UCN2/3 and CRF_2_ receptors still mediates as shown by others and us [30,31,32,33,34]. Urocortins are known to regulate vascular permeability [35]. In pregnancy, it has profound vasodilatory effects on the utero-placental and feto-placental circulations [36,37]. The placenta, an organ of fetal origin, is key for survival for the developing fetus inside a mother’s womb. During pregnancy, the placenta serves as an additional site for CRF synthesis and release [38,39] and CRF serves as a “placental clock” that determines the onset of labor [40,41]. The role of UCN2 and UCN3 in glucose homeostasis and diabetes is emerging [32,33,34,42,43], whereas CRF_2_, the predominant peripherally expressed receptor, is a key mediator of sexually dimorphic responses in diabetes phenotype [34]. The role of UCN1, UCN2, and CRF_2_ is also emerging in several gastrointestinal diseases, as discussed in later sections.

## 4. Gene Structure and Tissue Distribution of CRF and Urocortins

The human *CRH* gene is located on chromosome 8 and contains two exons. The human *UCN* (or *UCN1*) gene is located on chromosome 2 and contains two exons, and the entirety of the coding region is contained within the second exon. The *UCN2* gene is located on chromosome 3 and contains two exons. The least studied member of the CRF family is *UCN3*; it is located on chromosome 10 and consists of two exons. In mice, the *Crh* gene is located on chromosome 3 and consists of two exons. The *Ucn1* gene is located on chromosome 5 in mice and contains two exons. The *Ucn2* gene is located on chromosome 9 and contains one exon, and the mouse *Ucn3* gene is located on chromosome 13 and contains two exons. The genes for the *CRH* family of peptide hormones evolved after a series of gene duplication events (Figure 1A), and the phylogenetic evolution of CRF receptor paralogs is nicely summarized by Lovejoy et al. [2].

Interestingly, progeny from homozygous *Crh*^-/-^ mice (knockout of *Crh* gene) die within 12 h of birth due to lung dysplasia and atrophy of the adrenal gland [6]. *Crh*^-/-^ mice display marked fetal glucocorticoid deficiency and show impaired, sexually dimorphic stress responses (Table 1) [5]. However, progeny from *Crh*^-/-^ mice can be rescued by exogenous glucocorticoid administration and once rescued, the pups develop into adults with normal growth, fertility, and longevity [5]. Bhargava et al. reported that the PVN-specific elimination of *Crh* using RNAi alters stress-induced levels of ACTH hormone, but it did not affect basal ACTH levels [44], confirming that PVN-specific CRF plays a key role in the initiation of stress responses, but it is redundant for housekeeping functions. In agreement with PVN-specific RNAi findings, hypothalamus-specific *Crh* knockout mice have decreased basal, diurnal, and stress-activated plasma corticosterone secretion as well as basal plasma ACTH levels. In addition, these mice show adrenal atrophy and have noticeably reduced anxiety-like behaviors [45]. Mice overexpressing *Crh*, both in the brain and peripheral tissues (*Crh*-OE or *Crh*-Tg), have increased hypothalamic CRF and circulating corticosterone levels and show a phenotype resembling Cushing’s syndrome [46,47,48,49]. Taken together, these data suggest that either a lack of or overexpression of CRF hormone results in impaired stress, immune responses, and altered gastrointestinal function [5,15,21,50,51,52].

Two *Ucn1* null (*Ucn1*^-/-^) mice lines have been developed independently [53,54]. *Ucn1*^-/-^ mice are fertile and do not show any gross overt phenotypic abnormality. Curiously, male progeny from one of the lines display significant increases in anxiety-like behavior [53], whereas the male progeny in the second line do not differ from their wild-type littermates with regards to anxiety-like behavior [54]. Both lines harbor deletion of the coding exon and used identical genetic background (mixed 129S/C57BL6), thereby confounding results and suggesting that either animal housing environment or the sex of the researcher (or both) contribute to anxiety-like behavior [63]. However, both lines of *Ucn1*^-/-^ mice (of both sexes) have significant auditory deficits and acoustic startle responses (Table 1). Plasma membrane Ca^2+^ ATPase pumps (PMCAs) are responsible for the extrusion of Ca^2+^ from neurons, glia, and other cell types and are regulated by glucocorticoids as well as stressors [19,20,64]. Mice null for *Pmca1* are embryonic lethal, whereas heterozygous *Pmca1* mice display no overt phenotype [65]. Interestingly, spontaneous mutations in the *Pmca2* gene (A→G transition) resulting in glycine-to-serine substitutions in a highly conserved region of the PMCA2 protein causes deafness and imbalance in mice [66]. In agreement with these findings, mice null for *Pmca2* are deaf and heterozygous mice have significant hearing loss [67]. PMCA1 expression is repressed by glucocorticoids in the hippocampus of male rats [20]. In addition, various stressors, such as restraint and cold stress, repress hippocampal PMCA1 expression [20]. The treatment of cultured hippocampal cells with glucocorticoids repress its expression and function, resulting in high intracellular Ca^2+^ levels [Ca^2+^]_i_ and neuronal cell death due to faulty Ca^2+^ extrusion mechanisms [19]. Thus, the loss of PMCA function results in elevated [Ca^2+^]_i_ in the inner ear canals, which contributes to hearing loss in these mice. UCN1 has been shown to modulate Ca^2+^ signaling via the activation of CRF receptors [68,69]. Taken together, these data suggest that Ca^2+^ signaling mediated by PMCAs might be defective in the auditory canals of *Ucn1*^-/-^ mice, explaining this phenotype. The elimination of UCN1 expression in the vasculature enhances the capillary leak and migration of immune cells [35], confirming its role in vasoregulation.

*Ucn2* null mutant (*Ucn2*^-/-^) mice are viable and fertile but show a metabolic phenotype suggesting a role for UCN2 in glucose homeostasis [55]. Furthermore, the exogenous administration of UCN2 improves glucose clearance in diabetic male mice and acts as an insulin sensitizer in the skeletal muscles of obese male mice [32,33]. *Ucn3* null mice (*Ucn3*^-/-^) are also viable and fertile, and similar to UCN2, UCN3 is involved in the regulation of glucose homeostasis [57], as characterized using male mice null for *Ucn3*. Double and triple knockouts of urocortins are also reported [6,58]. These mice are viable (Table 1), but they reveal a key role of urocortins in stress recovery [6]. However, these knockout studies do not report an exhaustive characterization of morphology and function of all organs, and it is possible that urocortins knockouts may have impaired cardiac, liver, and reproductive functions. The readers are also referred to excellent reviews that summarize phenotypes for gene knockouts of the CRF family of peptide hormones and their two receptors [70,71,72].

**Figure 3:**
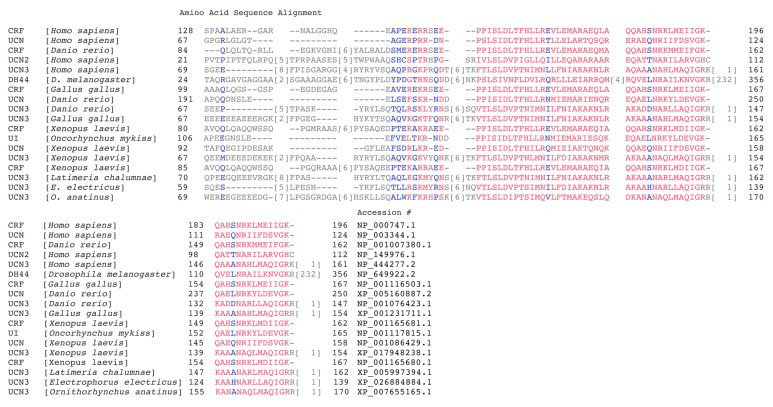
A comparison of examples of primary amino acid sequence from each group of CRF and urocortin precursor peptides found in the Protein data bank and generated using the multiple alignment tool from NCBI Blast. Red and blue are alignment columns with no gaps, whereas grey residues are with gaps. Highly conserved amino acids are shown in red and less conserved amino acids are shown in blue based on the residues’ relative entropy thresholds.

Both CRF and UCN1 are secreted as pro-peptide precursors that are 196 and 122 amino acids long, respectively [73]. The mature CRF peptide in humans is a 41-amino acid peptide hormone that is most closely related to UCN1, a 40-amino acid peptide, sharing approximately 45% amino acid identity with human and rat sequences. The human UCN2 has a size of 38 amino acids, sharing 38% amino acid identity with rat CRF and 42% amino acid identity with rat UCN1. The UCN3 consists of 38 amino acids and shares 26% amino acid identity with CRF and 34% amino acid identity with UCN1. Human CRF and all three urocortins also share homology with CRF and urocortins found in all other classes of vertebrata that include amphibian (frog), actinopetergyii (zebra fish and teleost), and avian (chicken) (Figure 3).

CRF is found in the parvocellular PVN of the hypothalamus, central nucleus of the amygdala, the bed nucleus of the stria terminalis (BNST), and the hindbrain regions in the central nervous system, amongst other regions [1,2,3]. The medial BNST regulates sexual behavior, whereas the lateral BNST regulates stress-related functions. In rats, no differences between the sexes are seen in the distribution of immunoreactive (IR) vasoactive intestinal polypeptide neurons that are in close proximity to CRF-IR neurons [74]. In male rats, lesions of BNST significantly decrease CRF mRNA expression in the medial parvocellular PVN [75]. When exposed to a myriad of stressors, CRF expression shows sexually dimorphic responses in the rodent brains. CRF expression in the amygdala, but not BNST or the hippocampus, differs between male and female mice exposed to predator odor [76]. However, in heterozygous knock-in mice for glutamic acid decarboxylase (GAD67), the enzyme responsible for the conversion of glutamate to GABA, exposure to predator order increases CRF expression in the BNST of male but not female mice compared with mice exposed to control odor [76]. In response to acute restraint stress exposure, CRF mRNA expression increases in the BNST of male, but not female rats, whereas transcription factor c-fos mRNA expression increases in female rats alone [77]. Exposure to chronic variable mild stress results in increases in total CpG (cytosine-guanine nucleotides) methylation of *Crh* gene in female but not male rats [78].

In the periphery, CRF is expressed in the various regions of the gut, including the enteric neurons, skin, adrenal glands, and the placenta [2,3]. While CRF expression is reported in several peripheral tissues, it remains unclear if CRF is synthesized locally in peripheral cells or is transported from the brain via circulation. RNAi experiments demonstrated for the first time that the knockdown of CRF in gut tissue severely decreased the expression of CRF-IR in the enteric neurons and the mucosa [8], suggesting local transcription and translation in these cell types. The gut-specific knockdown of CRF also prevents toxin-induced inflammation and demonstrates a role for locally transcribed and synthesized CRF and UCN2 in the regulation of both basal and stress-induced gut motility [8,9]. UCN1 is predominantly expressed in the Edinger Westphal and the suraoptic nuclei in the brain. In addition, UCN1-IR is also reported in the BNST [79]. In response to maternal separation, UCN-IR neurons show sexually dimorphic expression of c-Fos that is concomitant with dampening of the corticosterone responses in female but not male rats [79]. In the periphery, UCN1 is found in the intestine, cardiac myocytes, thymus, skin, spleen, and immune cells. UCN2 is expressed in the hypothalamus, brainstem, and spinal cord. In the periphery, it is found along the gastrointestinal tract, heart, blood cells, and adrenal gland [80,81,82]. UCN3 is found in the hypothalamus and amygdala, the intestine, and the pancreas [81,83,84,85]. Urocortins are distributed throughout the neuroaxis and peripheral tissues [7,8,33,36,42,61,80,83,86,87,88,89,90,91,92,93] with UCN1 being abundantly expressed in early and late gestational tissues [94,95,96].

## 5. Gene Structure and Tissue Distribution of CRF Receptors

CRF receptors also evolved via a series of unknown duplication events [1,2] and belong to the class B family of GPCRs (Figure 1C). CRF_1_ and CRF_2_ are products of two separate genes [97,98] and have several splice variants expressed in various central and peripheral tissues. In humans, CRF_1_ is encoded by the *CRHR1* gene, which contains 14 exons across 20 kb and is located on the long arm of chromosome 17. The entire length of *CRHR1* is approximately 51.55 kb. The entire gene product is termed CRF_1_-β, which is a 444-amino acid protein receptor that is involved in signaling properties and impaired agonist binding [97]. Removing exon 6—a section of *CRHR1* that contains the information for a 29-amino acid peptide—from the mRNA results in the translation of CRF_1_-α, which is a 415-amino acid long receptor that is the predominant functional receptor isoform. CRF_1_-α has high binding affinities for CRF and UCN1, facilitating their actions, and it is expressed in specific central and peripheral tissues throughout the body (Figure 2). CRF_1_ is also known to have six other subtypes, as a result of extensive alternative splicing; these are classified “c–h” and are present in humans and rodents, with several subtypes detailed to be nonfunctional. The subtypes all have a deletion of exon 6 and one or more exon excision, with the exception of subtype h, which has an insertion of a cryptic exon in the space separating exon 5 and 6. In mice, 13 exons are reported for the *Crhr1* gene. CRF_1_ knockout mice (*Crhr1*^-/-^) are known to display chronic glucocorticoid deficiency as a result of interference with the HPA axis [59]. In addition, *Crhr1*^-/-^ mice exhibit reduced anxiety-related behavior. Whereas heterozygous *Crhr1* mice are fertile, progeny from the homozygous *Crhr1*^-/-^ cross die within 48 h after birth with lung dysplasia [59]. The promoter region of the *Crhr1* gene harbors several binding sites for transcription factors such as SP1 and AP1 and hormone response elements that bind glucocorticoid, progesterone, and estrogen receptors [99].

CRF_2_, which is encoded by the *CRHR2* gene, has three variants: α, β, and γ. The human *CRHR2* gene is 50 kb in length and contains 15 exons. Whereas CRF_2_-α and CRF_2_-β are found in both humans and rodents, CRF_2_-γ is only found in the limbic region of the human brain. The three functional subtype mRNAs are made by utilizing an alternate 5′ exon 1 that connects onto a common set of exons. α, β, and γ variants have the same C-terminus and transmembrane domains [100]. The differences emerge in their N-terminal extracellular domains. CRF_2_-α has 34 amino acids at the N terminus and CRF_2_-β has 61 amino acids at the N terminus, whereas CRF_2_-γ has only 20. In summary, the α variant is a 411-amino acid receptor, the β variant is a 431-amino acid receptor, and the γ variant is a 397-amino acid receptor [101,102]. CRF_2_-γ shows no significant homology to the other two variants [101]. The three functional subtypes of CRF_2_ all contain different promoters and splicing control regulation [100]. Moreover, the three variants’ diverse expression is connected to the regulation of expression in distinct tissues. Two independent lines for CRF_2_ knockout mice (*Crhr2*^-/-^) have been generated [60,61]. Male *Crhr2*^-/-^ mice are hypertensive, have increased ACTH and glucocorticoid release, i.e., are hypersensitive to the HPA axis-mediated stress responses, and show increased anxiety-like behavior [60,61]. The promoter region of *Crhr2* gene also harbors several binding sites for SP1 as well as for glucocorticoid and estrogen receptors [100,103]. *Crhr1/Crhr2* double knockout mice display sexually dimorphic HPA axis stress responses (Table 1): female mice have decreased anxiety-like behavioral responses, whereas male mice show increased anxiety-like behavior [62].

**Figure 4:**
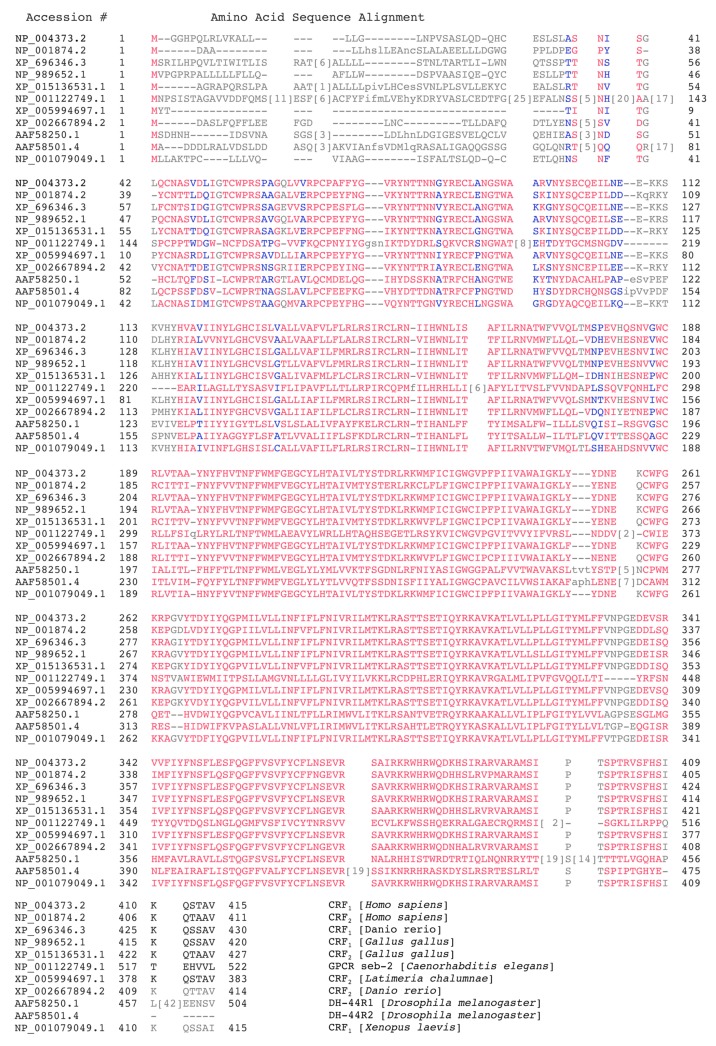
A comparison of examples of primary amino acid sequence from CRF receptors and their closely related GPCRs, seb-2, and diuretic hormone 44 receptors found in the Protein data bank and generated using the multiple alignment tool from NCBI Blast. Red and blue are alignment columns with no gaps, whereas grey residue are aligned with gaps. Highly conserved amino acids are shown in red, and less conserved are shown in blue based on the residues’ relative entropy thresholds.

CRF_1_ and CRF_2_ share 70% amino acid sequence similarity overall and nearly 80% identity in their transmembrane and intracellular domains. However, at the amino acid level, CRF_1_ and CRF_2_ exhibit low homology at the N-terminus: approximately 47% [100]. CRF receptors evolved from an ancestral GPCR seb-2 that is found in worms, including *C. elegans* (Figure 4). The receptors diverged after a gene duplication event to diuretic hormone (DH) receptors. Two DH44 receptors are reported in the fruit fly, *Drosophila melanogaster*, and other orthologs are found throughout the phyla (Figure 1C). Amino acid sequence alignment reveals a high sequence identity between DH44 receptors CRF_1_ and CRF_2_ (Figure 4). The two receptors are generally distinguished by their location in the central nervous system [2] and peripheral nervous system, in addition to their ability to differentially bind UCN1-3 and CRF [104]. CRF_1_ protein expression is sparse and not sexually dimorphic at postnatal days 0–21 in the PVN, but adult male mice show higher CRF_1_ levels in the PVN than female mice [105]. In rats, the sexually dimorphic expression of CRF receptors is seen in several brain regions with no two brain areas being alike [106]. CRF_1_ binding in various brain regions is similar in both male and female juvenile rats, but it is higher in the cingulate cortex of adult female rats compared with male rats. In contrast, CRF_2_ binding is greater in the BNST of adult male compared with female rats, irrespective of age [106].

Gonadectomy in young mice (6 weeks old) results in a significant decrease in CRF_1_-IR cells in the PVN of male but not female mice. CRF_1_ cells co-express estrogen receptor alpha (ERα) and androgen receptors, suggesting that circulating gonadal sex hormones differentially regulate CRF_1_ expression and function. Restraint stress induces a sexually dimorphic pattern of neural activation in PVN with male mice showing higher CRF_1_-IR compared with female mice [105]. In gonadectomized young adult male mice, treatment with an androgen receptor agonist increases the expression of CRF_2_ mRNA expression in various limbic regions and the lateral septum [103]. Thus, androgens might dampen stress responses and reduce anxiety-like behaviors in males of a species via mechanisms that involve increasing the expression of CRF_2_. In monogamous prairie voles, significantly higher CRF_2_ binding is reported in the BNST of males compared with females, suggesting a role for CRF_2_ in pair bonding [107]. The evolution, expression, distribution, and functions of CRF, urocortins, and their receptors are addressed in several excellent reviews and the references therein [1,2,3,15,52,70,71,72].

## 6. Agonist-Induced CRF Receptor-Regulatory Protein Supercomplexes

In vitro ligand-binding studies reveal that CRF binds with high affinity to CRF_1_ and has very low affinity for CRF_2_ [104]. UCN1 binds with 10–40-fold higher affinity to CRF_1_ than CRF and shows equal binding affinities for both CRF_1_ and CRF_2_ (Figure 2). UCN2 and UCN3 bind exclusively to CRF_2_, with UCN3 exhibiting a higher affinity than UCN2 [104,108]. CRF_1_ binds with higher affinity to UCN1 than CRF; however, this binding affinity does not translate to signaling efficacy [68]. Meanwhile, CRF_1_ does not internalize and signal when stimulated with UCN2 or UCN3, whereas, in the presence of CRF_2_, the two receptors heteromerize, internalize, and signal in response to these ligands [69].

The current ligand–receptor complex model is one in which the C-terminal domain of CRF binds to the N-terminal domain of the CRF_1_ receptor. The N-terminus can then bind to the J-domain formed by the seven-transmembrane receptor that couples to various G proteins to signal [109,110]. The receptors’ ligand selectivity is induced by the presence of a single nucleotide difference in the extracellular N-terminal domains of the receptors CRF_1_ and CRF_2_ [108]. The agonist selectivity of CRF_1_ and CRF_2_ can be attributed to the variance in homology at the N-terminus, which is involved in ligand binding [108] as well as differences in amino acid residues within the four agonists [104]. When exposed to excessive concentrations of agonist or ligand, CRF_1_ receptor internalization and desensitization occurs, which is a response that is common to most GPCRs. G-protein coupled receptor kinase 3 phosphorylates the CRF_1_ receptor, which is followed by the βarrestin2 protein trafficking the CRF_1_ receptor from the membrane into the cell via endocytosis [111], although the association of CRF receptors with βarrestins is very transient and unlike that of class B GPCRs [68]. This process represents the internalization of the CRF_1_ receptors, which are later marked for degradation or sent back to the plasma membrane i.e., recycled. CRF_1_ receptors traffics and signal differentially when bound to CRF versus UCN1 [68]. When bound to CRF, CRF_1_ receptors endocytose and recycle to the cell surface via fast recycling Rabs and are resensitized within 2–4 h of ligand stimulation. In contrast, UCN1-bound CRF_1_ receptors traffic via slower recycling Rabs, and approximately 20% of the receptors are targeted for degradation. Furthermore, endothelin-converting enzyme 1 is involved in the dissociation of UCN1-bound CRF_1_ receptors that couple to Gq but not to Gs [68].

Both CRF receptors harbor either pseudo or cleavable signal peptides [69,112,113,114,115,116]. These signal peptides are probably cleaved in a cell- or context-specific manner. For example, the signal peptide of CRF_1_ is reportedly cleaved in one study, but the N-terminal tag of CRF_1_ is not cleaved in others. CRF_2_-α is shown to harbor a pseudo signal peptide that can be converted to a cleavable one [113], whereas CRF_2_-β harbors a cleavable signal peptide [69]. Trafficking and signaling of CRF receptors has been described in several excellent reviews previously [109,110].

Upon ligand stimulation, GPCRs or associated proteins are post-translationally modified and can exist as super complexes in lipid rafts [117]. CRF_2_ binds at least four different ligands in its N-terminal extracellular domain and can potentially bind other ancillary proteins via its C-terminal PDZ-domain or intracellular 3 (i3) loop. Several cytoskeleton-associated proteins interact with the i3 loop of GPCRs including F-actin and filamin A [118,119,120]. Yeast two-hybrid screens have been extensively used to identify such interactions, but a major caveat of such a screen is that it detects only binary interactions between two binding partners. CRF_2_ interacts with F-actin and uses the F-actin cytoskeleton to traffic from the endoplasmic reticulum to the cell surface [69]. In conditions where the polymerization of F-actin was prevented, CRF_2_ receptors fail to reach the plasma membrane, whereas CRF_1_ receptor trafficking to the plasma membrane remains unaffected, suggesting that the two receptors use divergent pathways to travel to the cell membrane from internal compartments [69].

CRF_1_ can form homo-oligomers at the plasma membrane and in intracellular compartments [116,121]. However, as a result of pseudo signal peptide existence in the receptor, CRF_2_-α acts as a monomer [116]. The configuration of heteromers of CRF receptors with other GPCRs has been suggested to regulate the integration of various systems, such as HPA activation and drug abuse, for instance. Direct allosteric interaction among CRF_1_ and vasotocin VT2 receptor (VT2R) receptors is suggested in studies, using heterodimerization as a way to explain CRF potentiation by arginine vasotocin [122]. CRF_1_ is known to hetero(di)merize with vasopressin receptor V1b to mediate synergistic actions of vasopressin and CRF [123]. As a heteromeric partner of serotonin receptor, 5HT_2_R, CRF_1_ responds to serotonin, but not CRF, to signal via IP3 [124]. CRF modulatory action in the ventral tegmental area has been linked to the CRF_1_ activation of certain signaling pathways (phospholipase C, protein kinase A and C (PLC, PKA, and PKC)) in addiction studies and evidence of CRF_2_-α and dopamine receptor 1 interaction in HEK293T cells provides corroboration of joint action between dopamine neurotransmitter and CRF in neurons [121]. The two CRF receptors have the ability to form heteromeric complexes in association with regulatory proteins. CRF_1_ and CRF_2_-β can combine with other proteins to create heteromeric complexes in HEK293 cells co-expressing both receptors and in vivo in the pancreases of male mice [69]. These heteromeric complexes provide insight into the diverse and nuanced function achieved by members of the CRF system.

The sex-specific trafficking of CRF_1_ receptors is reported in one study. This study used brain slices to show that in response to swim stress, male rats have higher CRF_1_ receptor internalization and association with βarrestin2, whereas female rats do not [125]. In contrast to male rats, stressed female rats have a higher proportion of receptors on the plasma membrane of neurons in the locus-coeruleus (LC)-norepinephrine system than the unstressed rats, indicating the opposite trafficking pattern in females. The LC-norepinephrine system is the part of the brain thought to be in charge of regulating hyperarousal in patients with stress-related psychiatric disorders. A probable explanation for these sex-specific trafficking directions in internalization suggests differences in the relationship between CRF_1_ receptors and βarrestin2 [111,125]. Thus, studies addressing the sex and context-specific trafficking and signaling of CRF receptors will help in understanding the differential and nuanced effect of this system in a myriad of diseases that range from psychiatric to cardiovascular and from gastrointestinal and metabolic to reproductive diseases.

## 7. Sex-Specific Responses Mediated by Urocortins in Health and Disease

Urocortins are regulatory factors of the cardiovascular system, and the effects of UCNs in the periphery are largely mediated by the activation of CRF_2_. Studies suggest that the urocortins have the following effect on the cardiovascular system: vasodilation, cardioprotection against ischemia–reperfusion injury, and positive inotropic and lusitropic effects. Understanding the roles and mechanisms of UCNs are significant since they can be targets for treatment modalities for coronary heart disease, hypertension, and congestive heart failure [83,86,88,91,126,127,128,129]. UCNs have positive effects in the treatment of heart failure. UCN2 when injected peripherally acting via CRF_2_ raises left ventricular ejection fraction and cardiac output levels in the treatment of heart failure patients, corresponding with a decrease in vascular resistance [129]. UCNs also protect against ischemia as a result of a decrease in infarct size [88], as well as amelioration of the ventricular function by lessening myocardial damage and obstructing apoptosis pathways [127]. The pre-conditioning and post-conditioning of neonatal rat cardiomyocytes with UCN1 after a period of ischemia–reperfusion injury decreased apoptosis and necrosis, suggesting that UCN1 exerts cardioprotective effects [130].

The findings from a study that ascertained the effects of UCN2 in male rats with heart failure reveal that increased CRF_2_ receptor expression in the heart is correlated with a higher survival rate, without aggravating stress-related behavior [128]. The results from this aforementioned study are consistent with other studies showing that UCN2 and UCN1 in ventricular myocytes increase the rate and effectiveness of cardiac myocyte contractility [131,132]. UCNs serve to enhance cardiac myocyte contractility, since UCNs increase the concentration of intracellular calcium ions [129,132]. An increased concentration of Ca^2+^ in the cytosol of cardiac myocytes results in increased contraction of the heart. When CRF_2_ is inhibited, the inotropic and lusitropic effects of UCNs are removed. UCN2, the cognate ligand for CRF_2_ is known to improve cardiac function and recover the amplitude of Ca^2+^ transients by inhibiting the expression and interaction between store-operated Ca^2+^ channels, such as Ora1, and TRPC5 [133].

The sex differences in coronary risk and treatment are well documented [13], yet the biological mechanism is poorly understood. In a population-based prospective study with a sample size of 34,000 people, with a roughly even distribution of men and women, it was found that men are twice as likely as women to have a myocardial infarction, even after accounting for traditional risk factors such as blood pressure, serum lipid levels, and body mass index, among others [134]. The authors conclude that given the minor changes in incidence risk in myocardial infarction between the pre- and postmenopausal stage in women, with women still at lower incidence than men, it is unlikely that changes in female hormone levels influence the risk of myocardial infarction [134].

The differences in CRF receptor signaling and trafficking between men and women could partly explain how biological sex affects cardiovascular health and other diseases in which these receptors and their ligands are involved. Irrespective the tissue/organ, CRF_2_ expression appears to be protective and beneficial, whereas loss or inability to increase CRF_2_ expression leads to pathology. Should the effect of UCNs be sex-specific in cardiovascular tissue and CRF receptors be upregulated in premenopausal or cycling females compared to males, then sexual dimorphism in the CRF system may have significant implications. A broader understanding of the sex differences regarding CRF_2_ and the urocortins in the cardiovascular system and experiments that include female subjects are crucial to uncovering the underlying mechanisms.

## 8. Sex-Specific Differences in Psychiatric and Related Disorders

Psychiatric disorders such as post-traumatic stress disorder (PTSD), depression, and anxiety both have common origins in disrupted HPA axis and corticolimbic circuits. The hypersecretion of CRF and disruptions in processes downstream from the initial CRF are characteristic of many psychiatric disorders. The HPA axis is regulated by the CRF pathway, which when disturbed can affect how the stress response is both initiated and received. In humans, ACTH levels are increased further in women than in men, suggesting greater susceptibility to CRF. Therefore, the manipulation of CRF levels distresses the HPA axis more in women than in men [135]. The LC-norepinephrine system mentioned earlier is another target of the CRF pathway, and sex differences in LC neurons suggest that there are important considerations to be made in the treatment of stress-related psychiatric disorders. Patients suffering from psychiatric disorders such as PTSD and anxiety disorders experience hyperarousal, which is mediated by the LC-norepinephrine system. This system is often activated along with the HPA axis and at least partially regulated by CRF [136,137,138]. Animal studies show that female rats have more LC neurons than male rats, which could play a role in the differences in the manifestation of stress-related disorders [139,140]. LC dendrites appear to be more complex and extensive in female rats, with morphological analysis indicating longer and further radiating LC dendritic trees in females compared to males [141]. With denser, branching, and more complex dendrites, female rodents, and by extrapolation, human females are probably better equipped to process more emotional stimuli and initiate a greater arousal response.

CRF administration in the brain promotes grooming (anxiety-like relieving behavior) in both sexes, but at a greater magnitude in female than male mice. This discrepancy is greatest when ovarian hormones were highest in female mice, indicating a possible relationship between hormone levels and CRF-induced activation of grooming [142]. In another study that induced CRF overexpression in early development in mice, both sexes display anxiety-related behavior in adulthood, with the behavior being more pronounced in female mice. When stimulated with a secondary stressor in adulthood, female mice show avoidance regardless of history of CRF overexpression, whereas only male mice affected by CRF overexpression earlier show significant avoidance [143]. Studies show that neurons in the Edinger–Westphal nucleus co-express estrogen receptor (ERβ) and UCN1 immunoreactivity in both rat and mouse tissues [143]. mRNA levels of UCN1 in the Edinger–Westphal nucleus of male mice are approximately 10 times higher than in the Edinger–Westphal nucleus of female mice in diestrus and 1.6 times higher than in female mice in proestrus. Estrogen decreases the transcriptional activity of the UCN1 promoter via ERβ [144]. UCN1 immunoreactivity was also increased in the mid-brain region of male rats exhibiting alcohol preference behavior compared with controls, whereas UCN1 expression did not differ between control and alcohol-consuming female rats [145]. Others report a decrease in levels of UCN1 in pregnant compared with non-pregnant female rats [146].

In human studies, male suicide victims with major depression show higher UCN1 levels in the brains compared with healthy subjects, whereas no changes in UCN1 levels are noted between female suicide victims and control [147]. Interestingly, UCN1 and brain-derived nerve growth factor appear to be expressed in the opposite direction in a sex-specific manner in suicide victims with major depression. Single nucleotide polymorphisms (SNPs) in the *CRHR2* gene in human studies have been shown to associate with PTSD symptoms in women alone [148]. SNPs in the *CRHR2* gene are strongly associated with lifetime PTSD diagnosis in women but not men [148]. Corticolimbic circuitry is differentially activated more in women than in men, which could indicate why traumatic and/or stressful events have a greater effect. Women show increased susceptibility to stress disorders including PTSD and depression possibly due to changes in neuronal plasticity [149]. That same vulnerability is explicit in the role of CRF_2_ in reducing the effect of those stress disorders with the diminishing of symptoms associated with the *CRHR2* gene.

Genetic vulnerability for major depressive disorder is also been linked to the *CRHR1* gene [150]. In a population study of Han Chinese, three SNPs within the *CRHR1* gene were identified. Haplotype with alleles G-G-T, for respectively the SNPs of rs1876828, rs242939, and rs242941, is significantly over-represented in major depressive patients compared to healthy control, suggesting that the carriers of this haplotype might have an increased probability of developing major depression in Han Chinese population. In another study, the same three haplotype-tagged SNPs of *CRHR1*—rs242939, rs1876828, and rs242941—also associate with a genetic risk for depression during pregnancy and post-partum [151]. SNPs in *CRHR1* are shown to contribute to the development of irritable bowel syndrome (IBS) [152]. Three SNPs in *CRHR1* (rs110402, rs242924, and rs7209436) were studied and IBS was associated with the major allele for these SNPs. Studies have found a significant interaction between SNP rs110402 of the *CRHR1* gene and the environment (childhood trauma) regarding the development of depression in adults [153,154]. Subsequent studies show that the protective effect of the rs110402 A allele against developing depression after childhood trauma is observed in men but not in women [155]. Interestingly, women allelic carriers of rs110402 SNP did not have altered cortisol response to the dexamethasone/CRF test, but male rs110402 allele carriers show decreased cortisol responses compared to male GG genotype carriers. Thus, environment and biological sex display complex interaction for the development of psychiatric disorders in humans.

## 9. CRF System in Sex-Specific Regulation of Gut Function and Pathology

While epidemiological data suggest that functional gastrointestinal diseases are more prevalent in women than men, these diseases also present sex-specific symptoms [10]. However, only recently have studies focused on elucidating mechanisms that might explain some of these sex differences in disease manifestation [156]. Exogenous administration of CRF and urocortins in the brain can lead to stress-related alterations of gut motor function, and the injection of CRF antagonists can prevent the effects of stressors, which provides evidence for the significant role of CRF receptors in the regulation of stress-induced alterations in gastrointestinal motility. CRF initiates the inhibitory effect of gastric transit and motility by acting upon CRF_2_ [157,158]. Stressful episodes often precede flares associated with inflammatory bowel disease (IBD). In experimental animal models of colitis, a role for urocortins and CRF_2_ has been elucidated. Global knockout of the CRF_2_ receptor function or gut-specific elimination of CRF_2_ with RNAi exacerbates inflammation in experimental models of colitis [7,71,92]. Gut-specific elimination of CRF_2_, but not CRF_1_, skews tumor-necrosis factor-α (TNF-α) and extracellular signal-regulated kinase (ERK) signaling [92]. Intriguingly, the administration of UCN1 in male *Crhr2* heterozygous mice with colitis ameliorates inflammation and increases survival. In sharp contrast, in female *Crhr2* heterozygous mice, UCN1 administration increases mortality [92]. The increase in the survival and amelioration of colitis symptoms in *Crhr2* heterozygous male mice is attributed to UCN1′s ability to decrease levels of TNF-α, interleukin (IL)-6, and IL-1β as well as restore mitogen-activated protein kinase (MAPK) and heat shock protein 27 signaling in a sex-specific manner [92]. CRF signaling in the gut also acts upon mast cells to regulate inflammation as well as feeds back to the hypothalamus to regulate anxiety-like behavior in a rat model of functional dyspepsia [159]. In patients with diarrhea-predominant IBS, disease severity correlates with CRF_2_ receptors in B7+ mast cells/extracellular vesicles [160]. Mast cells are important targets as their pliability permits fast and selective immune responses as well as regulate gut permeability [161]. Mast cells harbor CRF receptors, and the two receptors mediate opposite signaling in immune cells [50]. 

While studies in human samples have demonstrated a role for the components of the CRF system in the regulation of immune responses, few have ascertained sex-specific actions of these hormone receptors. CRF regulates macrophage function by increasing lipopolysaccharide-controlled cytokine production in a CRF receptor-dependent manner [162,163]. In human studies, colons from IBD patients show altered expression levels of UCN1, UCN2, and CRF_2_ [87,92,164,165,166]. CRF_2_-IR is found in goblet and immune cells in the duodenum and colons of human subjects with levels of UCN1 and CRF_2_ increasing in male but not female patients with Crohn’s disease [92]. Furthermore, the spatiotemporal distribution of UCN1–CRF_2_ differs between male and female subjects, where co-localization is seen in gut samples from male but not female Crohn’s disease patients [92].

**Figure 5:**
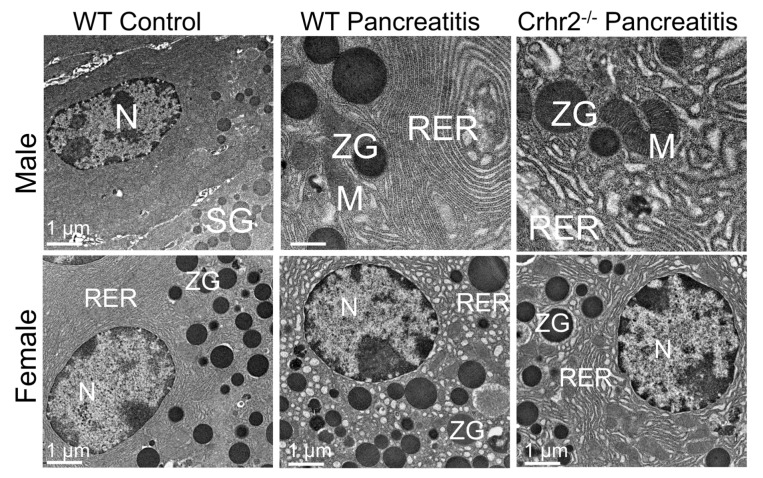
Electron micrographs showing representative pancreatic acinar cell sections from control and pancreatitis male and female mice. Endoplasmic reticulum (RER) shows whorling and the distortion of cisternae; mitochondrial (M) swelling is also evident. N: nucleus; SG: secretory granules; WT: wild-type; ZG: zymogen granules. Scale bar: 1 µM. Modified from Kubat et al. [93].

The sex-specific role of UCN1 and CRF_2_ is reported in the pathogenesis of pancreatic inflammation [93]. While treatment with caerulein induces pancreatitis in both male and female mice, after an identical dose of caerulein, C57BL6 female mice show less severe pancreatitis than C57BL6 male mice [93]. Female mice show less necrosis, zymogen granules, and vacuolization than male mice, but they have similar levels of edema and neutrophil infiltration as male mice [93]. In female *Crhr2*^-/-^, caerulein induces more severe inflammation compared to male mice [93]. Sex-specific differences in rough endoplasmic reticulum (RER) ultrastructure along with signaling in unfolded protein response and autophagy are also reported [93]. While pancreatic acinar cell ultrastructure from saline-treated male and female mice is very similar, the induction of pancreatitis resulted in more severe RER ultrastructure damage in the acinar cells of female compared with male mice (Figure 5, left versus middle panel). In male mice lacking a function CRF_2_ receptor (*Crhr2*^-/-^), pancreatitis causes severe and more dramatic distortion of RER cisternae, mitochondrial swelling, and unusual autophagic bodies compared to C57BL6 mice of both sexes as well as *Crhr2*^-/-^ female mice (Figure 5, top right panel). Interestingly, an in-between phenotype is seen in heterozygous *Crhr2* mice i.e., mice haploinsufficient for CRF_2_ receptor function. These findings suggest to us that cellular stress downregulates CRF_2_ receptors, putting an undue burden on organelles such as the RER and mitochondria. RER is the main organelle responsible for protein synthesis and quality control, whereas mitochondria regulate cellular respiration and oxidative stress. These damaged RER and mitochondria structure/function would explain the increase in accumulation of misfolded proteins, altered secretory capacity, and oxidative stress. If this ultrastructure damage occurs in other cell types as well, it would provide a logical explanation as to why stress exacerbates the accumulation of misfolded proteins and increases oxidative stress in other pathologies, such as neurological diseases. Interestingly, UCN1 ameliorates pancreatitis severity in male but not in female mice by restoring several of the above-mentioned signaling pathways. In another study, UCN2 decreases pancreatitis symptoms and inflammation via nuclear factor kappa-light-chain-enhancer of activated B cells (NF-κB) pathways [167].

Globally, 14 million more men are diagnosed with type 2 diabetes than women, despite more women being obese than men, as reflected by their body mass index measurements [168]. Interestingly, impaired glucose tolerance is more common in women than in men, independent of age. Stress is known to cause an elevation in blood glucose levels, diabetes, fatty liver, obesity, and metabolic syndrome [169]. Prospective longitudinal cohort studies spanning >10 years show that *perceived stress* increased the risk of developing diabetes in men by approximately 2-fold and had a 1.4 higher odds ratio than women. Perceived stress is also associated with increased insulin resistance. The same study reports that excessive work and work reward imbalance is also associated with a nearly 2-fold increased risk for diabetes in men but not women [170]. Short duration or poor initiation of sleep is also associated with an increased risk of diabetes in both men and women. A recent genome-wide association study of approximately 890,000 people with type 2 diabetes (DIAGRAM consortium) [171] identified several novel diabetes susceptibility loci including the stress receptor corticotropin-releasing factor receptor 2 (Crhr2). Thus, stress, whether emotional or physical, is highly associated with unhealthy lifestyle and behavior that includes eating behaviors, low exercise levels, smoking and alcohol abuse, and poor sleep.

Meta-analysis studies reveal that diabetic women are at 50% greater risk for cardiovascular-related mortality and 30% greater risk for stroke than diabetic men. In a clinical trial on patients with diabetes and vascular disease (ADVACE-ON), glucose control with gliclazide resulted in less frequently end-stage kidney disease and subgroup analysis by sex shows that the risk reduction was only significant in men [172]. Women also have a higher risk of experiencing severe hypoglycemia with insulin therapy. Female sex, statin use, smoking, diabetes duration, body weight, and initial HbA1c all predict earlier failure of any “dual” treatment [172]. Despite this mounting evidence of sex-differences in the manifestation of metabolic disease in humans, very little is known about the sex-differences in metabolic signaling pathways.

Recent evidence from experimental models of diabetes suggests that urocortin 2 and 3 (UCN2–3) might be involved in the regulation of insulin secretion and blood glucose levels [33,43]. Islets lacking UCN3 show increased insulin secretion [43] and the exogenous administration of UCN2 normalized glucose levels in two distinct murine models of diabetes [33] and increased glucose uptake in the skeletal muscles of obese mice [32]. Studies from our laboratory have examined sex-specific actions of CRF_2_ receptor in diabetes [34]. In this study, we find that *male Crhr2*^-/-^ and *Crhr2* haploinsufficient mice have worse glucose and insulin tolerance, microvesicular hepatic steatosis, and dyslipidemia than female *Crhr2* mice, strongly suggesting that the *Crhr2* locus modulates the sex-specific expression of multiple genes influencing diabetes and metabolic syndrome in humans and mice. In agreement with our observations, a systematic study that characterized the effects of sex and body weight on metabolic outcomes in C57BL/6 mice found that male mice had worse glucose clearance on a high-fat diet than female mice [173]. Furthermore, this study used nearly 300 mice per sex to characterize metabolic phenotype on breeder’s chow and high-fat diet (HFD) to create a reference database [173].

## 10. CRF System in Reproduction

In the brain, CRF mRNA expression in the PVN is reported to be higher in female than male rats [174]. This discrepancy is associated with the estrous cycle, with CRF expression highly correlating with sex hormone levels [174]. Higher rates of depression are noted after puberty and before menopause in women, further supporting the probability of ovarian hormones playing a role in susceptibility to stress-related psychiatric disorders. Studies find that estrogen promotes CRF expression in the PVN, whereas androgens inhibit CRF expression in rodents [175]. A human study shows that men with higher levels of estrogen than controls have a greater number of hypothalamic CRF neurons, but the predicted decrease in CRF neurons in women after menopause are not found, suggesting that other factors besides sex hormones are at play [176]. The sexually dimorphic BNST brain region also shows CRF-IR that is regulated by gonadal sex steroids in the female mice [177]. As discussed earlier, both estrogens and androgens modulate expression levels of CRF_1_ and CRF_2_ receptors in rodents of both sexes. While expression of both ligands and receptor follows cycling of estrogen levels, androgens appear to dampen stress responses and reduce anxiety-like behaviors in male rodents by increasing expression of CRF_2_ [103,105], which is consistent with our observations, vis-à-vis, a role for CRF_2_ in exerting protective effects in inflammation and metabolic stress in peripheral tissues [7,34,92,93].

The CRF system’s importance to maternal–fetal stress patterns and embryo implantation further emphasizes the role it plays, specifically in females. In pregnancies with preeclampsia, CRF levels are increased, whereas CRF binding protein levels are at a lower level than in normal pregnancies, indicating the possibility of a greater proportion of free CRF having an effect on the pathology of preeclampsia [178]. With generally higher concentrations of free CRF in normal pregnancies as well, those heightened levels are known to increase placental vasodilation. Fetal urocortin levels measured at term without labor are lower than those at term with labor and in preterm labor [94]. UCN1 also plays a similar role, increasing uterine contractility and inducing the production of ACTH and prostaglandin PGE2 to vasodilate the placenta [90]. UCN2 and UCN3 could be vital to the development of the trophoblast and the continuation of pregnancy, but increased concentrations in preeclampsia placentae could represent a reaction to oxidative stress [179].

UCN1 is important for embryo implantation [90,180,181]. UCN1 is abundantly expressed in early and late gestational tissues [179]. In pregnancy, UCN1 regulates vascular tone [36,37]. Reduced circulating levels of UCN1 are associated with increased uterine artery resistances and may regulate uterine artery tone [182]. Urocortin directly and indirectly triggers myometrial contractility, which induces uterine contractions [37,90]. We have shown involvement of UCN1 in regulation of microvascular permeability [35]. Others describe its potential role in preeclampsia [179]. Blood flow regulation is key for the overall health of the placenta and brain development in the fetus. Thus, impaired UCN1 levels may result in prenatal complications and have significant implications for adverse pregnancy outcomes, such as preterm birth, low body weight, and neonatal morbidity.

**Figure 6:**
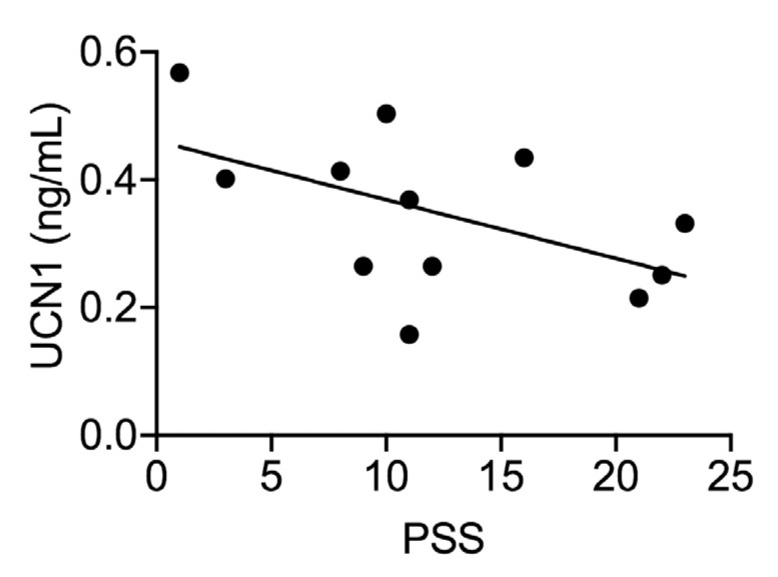
A scatterplot of the correlation between perceived stress score (PSS) and placental UCN1 levels. Analysis revealed that placental UCN1 levels were negatively correlated with PSS scores (r = 0.531, *p* = 0.07). Higher PSS scores indicate higher levels of stress experienced during the third trimester of pregnancy. Nulliparous women (n = 12) were recruited at the University of California San Francisco (UCSF) Mission Bay Hospital under UCSF’s institutional review board protocol # 16-10957. All women carried to term and completed PSS questionnaires between 37 and 40 weeks of gestation. Placentae were collected after delivery, and UCN1 levels were measured using ELISA.

Human studies that examined UCN1 in fetal cord blood or maternal plasma at birth also found significant relationships of UCN1 to risk of preterm birth, as well as associations with greater neonatal morbidity [183,184,185]. Two studies that used amniotic fluid acquired during midgestation found a minimal relationship of UCN1 to other stress measures or to birth outcomes [186,187]. In contrast, two other studies that examined levels in amniotic fluid found a relationship of higher UCN1 levels to preterm birth [188,189]. One study sampling maternal serum when labor was initiated did not find an association of UCN1 to risk of preterm birth [190]. Clearly, findings have not been consistent regarding the potential role of UCN1 in predicting adverse birth outcomes, which is probably due to variability in the timing of sample collection. However, it has been suggested that sampling in the second trimester (when the studies of amniotic fluid took place) may lead to less reliable results [187]. It has been suggested that the predictive values of the entire CRF system reach clinical reliability between 26 and 31 weeks of gestation [191]. Thus, research is needed that uses samples during the third trimester rather than midgestation to increase reliability in predicting outcomes. In agreement with these published observations about the role of UCN1, we have also found that placental/fetal UCN1 levels inversely correlate with perceived stress score (PSS) in a cohort of healthy pregnant women. As PSS increased, placental UCN1 levels decreased (Figure 6). Higher PSS indicates higher levels of stress, and lower UCN1 could lead to an insufficient vasodilation of the placenta. Excess vasoconstriction of the placenta is a known possible cause of preeclampsia, so combined with the indication that stress in mothers can affect the placenta during pregnancy, this suggested relationship warrants further exploration.

In the ovarian cycle, UCN1 targets steroidogenic luteal cells during luteal regression by binding to the CRF receptors. UCN1 is expressed in mid- and late-phase corpus luteum luteinized granulosa and thecal cells [96]. CRF and CRF_1_ receptor mRNA are found in higher levels in regressing corpus luteum than in the pregnant corpus luteum or the mid-luteal phase. These studies have not found evidence of UCN2 and UCN3 having a part in the ovary [192,193]. UCN1 mRNA is expressed during the endometrial phases of the menstrual cycle, with the highest concentrations of UCN1 and mRNA in the late secretory endometrial phase [194]. CRF and UCN1 could also play a role in endometriosis, as peritoneal endometriosis tissues with activated mast cells staining for both. This, along with heightened urocortin levels in endometriomas, indicates a relationship with the symptoms of endometriosis, and whether that correlation exists between low fertility, inflammation, fibrosis, or spontaneous abortions is still unknown [195].

The length of human gestation appears to be decided by the combination of the UCN1 from the fetus and the CRF from the placenta. In post-term pregnancy, CRF and UCN1 levels are lower than in term pregnancy, and the concentrations of both neuropeptides decrease as the induction-delivery interval increases [95]. Serum UCN1 measured from preterm deliveries and term deliveries were not significantly different, so maternal UCN1 does not seem to be a reliable marker [190]. Another study showed that low UCN1 levels in amniotic fluid at midterm are suggestive of preterm birth [188]. A recent study showed that a retroviral long terminal repeat transposon-like element 1B (THE1B) controls placental expression of CRF in humans and other anthropoid primates [196]. This element is not present in prosimians and non-primates, including rodents such as mice and rats. Placental CRF in turn influenced gestational age and birth timing in human pregnancies [196]. Placental, not hypothalamic (or brain), CRF expression is associated with gestational age in humans in this study. Taken together with results from several vertebrate experimental models and human studies, these data suggest that CRF and urocortins are key players in maintaining healthy pregnancy and birth outcomes.

## 11. Summary

**Figure 7:**
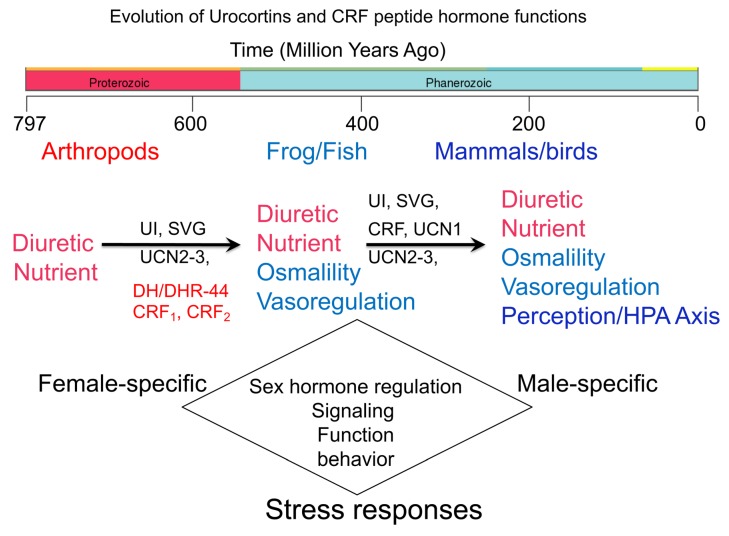
A summary schema showing the evolution of CRF and urocortins hormones and their functions. Diuretic hormones (DH44) and DH44 receptors in the fruit flies probably served as prototypes for urocortin 3 and 2, and CRF receptors, respectively, which first appeared in Actinoptergyii (fish) and Sarcoptergyii after a genome-wide duplication event nearly 400 million years ago. Distinct CRF, urotensin I (UI), urocortins, and sauvagine peptides first seemed to have appeared nearly 500 million years ago. The innervation of hypothalamic cells by CRF family members occurred in class Actinoptergyii along with appearance of urocortin-like peptides. The release of CRF into the portal circulation appeared in Sarcoptergyii, whereas UI-like and sauvagine peptides transitioned to being expressed in the skin in the amphibians. The innervation and descending of CRF fibers in the spinal cord seemed to have occurred nearly 290 million years ago [2]. As the peptides evolved, the functions appeared to have diversified to include osmo and vasoregulation, as well as regulation of HPA axis-mediated stress responses. By virtue of having sex hormone response elements in the promoter regions of all four hormones and two receptors, sex-specific differences in the expression and function mediated by the CRF system are noted.

The CRF family of peptide hormones and their two known CRF receptors regulate several physiological functions besides the well-characterized regulation of the HPA axis responses (Figure 7). Stressors in life are unavoidable. Stress response to physical, psychological, environmental or cellular stressors has two arms: *initiation* and *recovery*. CRF binding to CRF_1_ receptors is primarily responsible for initiating stress responses via the HPA axis and the release of glucocorticoids, whereas urocortins binding to peripherally expressed CRF_2_ receptors are responsible for the recovery response to stress and subjected to feedback regulation by glucocorticoids. We think that this system is in “over-drive”, and the inability of the system to return to homeostasis via mechanisms that largely involve CRF_2_ receptors puts undue burden on this system. The myriad of molecular and cellular signals and functions that this system orchestrates in multiple organs for a balanced stress response is an overwhelming task. Some of these signals and functions are shared between members of the two known biological sexes, whereas others are distinct. This ancient system’s role in regulating sexually dimorphic signaling responses in a plethora of pathophysiologies is emerging and will be key for developing therapeutic strategies that will be successful in a sex and gender-specific manner.

## Figures and Tables

**Table 1:** Phenotypic characteristics of transgenic modifications in genes for components of the CRF family of peptide hormones and receptors.

Genotype	Transgenic Modification	Gross Phenotype and Biological Characteristics
*Crh* ^-/-^	Knockout [5]	Progeny from homozygous *Crh*^-/-^ die within 12 h after birth due to lung hyperplasia. Can be rescued after intervention with exogenous glucocorticoids. Marked fetal glucocorticoid deficiency, but normal postnatal growth, fertile.
*Crh*-OE or *Crh*-Tg	Constitutive overexpression [46]	Elevated basal plasma corticosterone, normal basal ACTH, and HPA axis responses to stress.
*Crh*-OE or *Crh*-Tg	Overexpression [49]	Cushing’s syndrome-like symptoms. Increased hypothalamic CRF expression, increased circulating corticosterone and adrenocorticotrophic hormone (ACTH). Delayed and attenuated HPA axis responses to stress.
*Ucn1* ^-/-^	Knockout [53,54]	Significant auditory deficit and acoustic startle responses in both lines. Increased anxiety-like responses in one line, but not the other. No overt phenotypic abnormality. Fertile.
*Ucn2* ^-/-^	Knockout [55,56]	Metabolic phenotype suggesting role in glucose homeostasis. No other overt phenotypic abnormality. Fertile. Female, but not male knockout show increased basal ACTH, corticosterone rhythm, decreased fluid intake, and depression-like behavior.
*Ucn3* ^-/-^	Knockout [57]	Regulation of glucose homeostasis. No change in anxiety- or depression-like behaviors. No other gross phenotype. Fertile.
*Ucn1*^-/-^/*Ucn2^-/^*^-^	Double knockout [58]	Impaired recovery responses to stress. Fertile.
*Ucn1*^-/-^/*Ucn2^-/^*^-^/*Ucn3*^-/-^	Triple knockout [6]	Reveal urocortins’ role in stress recovery; increased anxiety-like behavior in response to stress. May have impaired cardiac and reproductive functions.
*Crhr1* ^-/-^	Knockout [59]	Low plasma corticosterone, impaired HPA axis responses, decreased anxiety-like responses. Progeny from heterozygous mice are fertile and without overt phenotype, but male progeny show 15% mortality. Progeny from homozygous female dam die 2 days after birth. Adrenal gland atrophy.
*Crhr2* ^-/-^	Knockout [60,61]	Hypertensive, increased anxiety-like behavior, hypersensitive to HPA axis stress responsive, fertile, no other overt phenotype.
*Crhr1^-/-^/Crhr2* ^-/-^	Double knockout [62]	Sexually dimorphic stress responses: female mice have reduced anxiety-like behavior, whereas male mice show increased anxiety-like behavior.

## References

[1] Lovejoy D.A., Balment R.J. (1999). Evolution and physiology of the corticotropin-releasing factor (CRF) family of neuropeptides in vertebrates. Gen. Comp. Endocrinol..

[2] Lovejoy D.A., Chang B.S.W., Lovejoy N.R., Del Castillo J. (2014). Molecular Evolution of GPCRS: CRH/CRH receptors. J. Mol. Endocrinol..

[3] Smith S.M., Vale W. (2006). The role of the hypothalamic-pituitary-adrenal axis in neuroendocrine responses to stress. Dialog. Clin. Neurosci..

[4] Kumar S., Stecher G., Suleski M., Hedges S.B. (2017). TimeTree: A Resource for Timelines, Timetrees, and Divergence Times. Mol. Boil. Evol..

[5] Muglia L., Jacobson L., Dikkes P., Majzoub J.A. (1995). Corticotropin-releasing hormone deficiency reveals major fetal but not adult glucocorticoid need. Nature.

[6] Neufeld-Cohen A., Tsoory M.M., Evans A.K., Getselter D., Gil S., Lowry C.A., Vale W.W., Chen A. (2010). A triple urocortin knockout mouse model reveals an essential role for urocortins in stress recovery. Proc. Natl. Acad. Sci. USA.

[7] Chang J., Adams M.R., Clifton M.S., Liao M., Brooks J.H., Hasdemir B., Bhargava A. (2011). Urocortin 1 modulates immunosignaling in a rat model of colitis via corticotropin-releasing factor receptor 2. Am. J. Physiol. Liver Physiol..

[8] la Fleur S.E., Wick E.C., Idumalla P.S., Grady E.F., Bhargava A. (2005). Role of peripheral corticotropin-releasing factor and urocortin II in intestinal inflammation and motility in terminal ileum. Proc. Natl. Acad. Sci. USA.

[9] Liu S., Chang J., Long N., Beckwith K., Talhouarne G., Brooks J.J., Qu M.-H., Ren W., Wood J.D., Cooper S. (2015). Endogenous CRF in rat large intestine mediates motor and secretory responses to stress. Neurogastroenterol. Motil..

[10] Prusator D.K., Chang L. (2017). Sex-Related Differences in GI Disorders. Handb. Exp. Pharmacol..

[11] Yehuda R., Hoge C.W., McFarlane A.C., Vermetten E., Lanius R.A., Nievergelt C.M., Hobfoll S.E., Koenen K.C., Neylan T.C., Hyman S.E. (2015). Post-traumatic stress disorder. Nat. Rev. Dis. Primers..

[12] Hajar R. (2016). Framingham Contribution to Cardiovascular Disease. Heart Views.

[13] Maas A.H., Appelman Y.E. (2010). Gender differences in coronary heart disease. Neth. Heart J..

[14] Berczi I.A. (2016). An Update on Neural Regulators of the Hypothalamic–Pituitary–Adrenal Axis. Insights to Neuroimmune Biology.

[15] Sapolsky R.M., Romero L.M., Munck A.U. (2000). How do glucocorticoids influence stress responses? Integrating permissive, suppressive, stimulatory, and preparative actions. Endocr. Rev..

[16] Stephens M.A., Wand G. (2012). Stress and the HPA axis: Role of glucocorticoids in alcohol dependence. Alcohol. Res..

[17] Gray J.D., Kogan J.F., Marrocco J., McEwen B.S. (2017). Genomic and epigenomic mechanisms of glucocorticoids in the brain. Nat. Rev. Endocrinol..

[18] Panettieri R., Schaafsma D., Amrani Y., Koziol-White C., Ostrom R., Tliba O. (2019). Non-genomic Effects of Glucocorticoids: An Updated View. Trends Pharmacol. Sci..

[19] Bhargava A., Mathias R.S., McCormick J.A., Dallman M.F., Pearce D. (2002). Glucocorticoids prolong Ca(2+) transients in hippocampal-derived H19-7 neurons by repressing the plasma membrane Ca(2+)-ATPase-1. Mol. Endocrinol..

[20] Bhargava A., Meijer O.C., Dallman M., Pearce D. (2000). Plasma Membrane Calcium Pump Isoform 1 Gene Expression Is Repressed by Corticosterone and Stress in Rat Hippocampus. J. Neurosci..

[21] Gurfein B.T., Hasdemir B., Milush J.M., Touma C., Palme R., Nixon D.F., Darcel N., Hecht F.M., Bhargava A. (2017). Enriched environment and stress exposure influence splenic B lymphocyte composition. PLoS ONE.

[22] Hiller-Sturmhofel S., Bartke A. (1998). The endocrine system: An overview. Alcohol. Health Res. World.

[23] Cannon W.B. (1929). Organization for physiological homeostasis. Physiol. Rev..

[24] Goldstein D.S. (2010). Adrenal responses to stress. Cell Mol. Neurobiol..

[25] Selye H. (1976). Stress without Distress. Psychopathology of Human Adaptation.

[26] Godoy L., Rossignoli M.T., Delfino-Pereira P., Garcia-Cairasco N., Umeoka E. (2018). A Comprehensive Overview on Stress Neurobiology: Basic Concepts and Clinical Implications. Front. Behav. Neurosci..

[27] Arase K., York D.A., Shimizu H., Shargill N., Bray G.A. (1988). Effects of corticotropin-releasing factor on food intake and brown adipose tissue thermogenesis in rats. Am. J. Physiol. Metab..

[28] Heinrichs S., Cole B.J., Pich E.M., Menzaghi F., Koob G.F., Hauger R.L. (1992). Endogenous corticotropin-releasing factor modulates feeding induced by neuropeptide Y or a tail-pinch stressor. Peptides.

[29] Spina M., Merlo-Pich E., Chan R.K.W., Basso A.M., Rivier J., Vale W., Arends M.A. (1996). Appetite-Suppressing Effects of Urocortin, a CRF-Related Neuropeptide. Science.

[30] Fatima A., Andrabi S., Wolf G., Engelmann M., Spina M.G. (2012). Urocortin 1 administered into the hypothalamic supraoptic nucleus inhibits food intake in freely fed and food-deprived rats. Amino Acids.

[31] Yakabi K., Noguchi M., Ohno S., Ro S., Onouchi T., Ochiai M., Takabayashi H., Takayama K., Harada Y., Sadakane C. (2011). Urocortin 1 reduces food intake and ghrelin secretion via CRF(2) receptors. Am. J. Physiol. Metab..

[32] Borg M.L., Massart J., Schönke M., Barbosa T.D.C., Guo L., Wade M., Alsina-Fernandez J., Miles R., Ryan A., Bauer S. (2019). Modified UCN2 Peptide Acts as an Insulin Sensitizer in Skeletal Muscle of Obese Mice. Diabetes.

[33] Gao M.H., Giamouridis D., Lai N.C., Walenta E., Paschoal V.A., Kim Y.C., Miyanohara A., Guo T., Liao M., Liu L. (2016). One-time injection of AAV8 encoding urocortin 2 provides long-term resolution of insulin resistance. JCI Insight.

[34] Paruthiyil S., Hagiwara S.-I., Kundassery K., Bhargava A. (2018). Sexually dimorphic metabolic responses mediated by CRF2 receptor during nutritional stress in mice. Boil. Sex. Differ..

[35] Cureton E.L., Ereso A.Q., Victorino G.P., Curran B., Poole D.P., Liao M., Harken A.H., Bhargava A. (2009). Local secretion of urocortin 1 promotes microvascular permeability during lipopolysaccharide-induced inflammation. Endocrinology.

[36] Borges L.E., Bloise E., Cruz C.D., Galleri L., Apa R., Petraglia F., Reis F.M. (2015). Urocortin 1 expression and secretion by human umbilical vein endothelial cells: In vitro effects of interleukin 8, interferon γ, lipopolysaccharide, endothelin 1, prostaglandin F-2α, estradiol, progesterone and dexamethasone. Peptides.

[37] Clifton V.L., Qing G., Murphy V., Schwartz J., Madsen G., Smith R. (2000). Localization and Characterization of Urocortin during Human Pregnancy. Placenta.

[38] Challis J.R.G., Matthews S.G., Gibb W., Lye S.J. (2000). Endocrine and paracrine regulation of birth at term and preterm. Endocr. Rev..

[39] Chrousos G.P., Torpy D.J., Gold P.W. (1998). Interactions between the hypothalamic-pituitary-adrenal axis and the female reproductive system: Clinical implications. Ann. Intern. Med..

[40] Thomson M. (2012). The physiological roles of placental corticotropin releasing hormone in pregnancy and childbirth. J. Physiol. Biochem..

[41] Wadhwa P.D., Garite T.J., Porto M., Glynn L., Chicz-DeMet A., Dunkel-Schetter C., Sandman C.A. (2004). Placental corticotropin-releasing hormone (CRH), spontaneous preterm birth, and fetal growth restriction: A prospective investigation. Am. J. Obstet. Gynecol..

[42] Jamieson P.M., Cleasby M.E., Kuperman Y., Morton N.M., Kelly P.A.T., Brownstein D.G., Mustard K.J., Vaughan J.M., Carter R.N., Hahn C.N. (2011). Urocortin 3 transgenic mice exhibit a metabolically favourable phenotype resisting obesity and hyperglycaemia on a high-fat diet. Diabetologia.

[43] Van Der Meulen T., Donaldson C.J., Caceres E., Hunter A.E., Cowing-Zitron C., Pound L.D., Adams M.W., Zembrzycki A., Grove K.L., Huising M. (2015). Urocortin3 mediates somatostatin-dependent negative feedback control of insulin secretion. Nat. Med..

[44] Bhargava A., Dallman M.F., Pearce D., Choi S. (2004). Long double-stranded RNA-mediated RNA interference as a tool to achieve site-specific silencing of hypothalamic neuropeptides. Brain Res. Protoc..

[45] Zhang R., Asai M., Mahoney C.E., Joachim M., Shen Y., Gunner G., Majzoub J.A. (2016). Loss of hypothalamic corticotropin-releasing hormone markedly reduces anxiety behaviors in mice. Mol. Psychiatry.

[46] Groenink L., Dirks A., Verdouw P.M., Schipholt M.L., Veening J.G., Van Der Gugten J., Olivier B. (2002). HPA axis dysregulation in mice overexpressing corticotropin releasing hormone. Boil. Psychiatry.

[47] Lu A., Steiner M.A., Whittle N., Vogl A.M., Walser S.M., Ableitner M., Refojo D., Ekker M., Rubenstein J.L., Stalla G.K. (2008). Conditional mouse mutants highlight mechanisms of corticotropin-releasing hormone effects on stress-coping behavior. Mol. Psychiatry.

[48] Nakayama S., Nishiyama M., Iwasaki Y., Shinahara M., Okada Y., Tsuda M., Okazaki M., Tsugita M., Taguchi T., Makino S. (2011). Corticotropin-releasing hormone (CRH) transgenic mice display hyperphagia with increased Agouti-related protein mRNA in the hypothalamic arcuate nucleus. Endocr. J..

[49] Stenzel-Poore M.P., Cameron V.A., Vaughan J., Sawchenko P.E., Vale W. (1992). Development of Cushing’s syndrome in corticotropin-releasing factor transgenic mice. Endocrinology.

[50] D’Costa S., Ayyadurai S., Gibson A.J., Mackey E., Rajput M., Sommerville L.J., Wilson N., Li Y., Kubat E., Kumar A. (2018). Mast Cell CRF2 Suppresses Mast Cell Degranulation and Limits the Severity of Anaphylaxis and Stress-Induced Intestinal Permeability. J. Allergy Clin. Immunol..

[51] Santos J., Saunders P.R., Hanssen N.P.M., Yang P.-C., Yates D., Groot J.A., Perdue M.H. (1999). Corticotropin-releasing hormone mimics stress-induced colonic epithelial pathophysiology in the rat. Am. J. Physiol. Content.

[52] Tache Y., Perdue M.H. (2004). Role of peripheral CRF signalling pathways in stress-related alterations of gut motility and mucosal function. Neurogastroenterol. Motil..

[53] Vetter D.E., Li C., Zhao L., Contarino A., Liberman M.C., Smith G.W., Marchuk Y., Koob G.F., Heinemann S.F., Vale W. (2002). Urocortin-deficient mice show hearing impairment and increased anxiety-like behavior. Nat. Genet..

[54] Wang X., Su H., Copenhagen L.D., Vaishnav S., Pieri F., Shope C.D., Brownell W.E., De Biasi M., Paylor R., Bradley A. (2002). Urocortin-Deficient Mice Display Normal Stress-Induced Anxiety Behavior and Autonomic Control but an Impaired Acoustic Startle Response. Mol. Cell. Boil..

[55] Chen A., Brar B., Choi C.S., Rousso D., Vaughan J., Kuperman Y., Kim S.N., Donaldson C., Smith S.M., Jamieson P. (2006). Urocortin 2 modulates glucose utilization and insulin sensitivity in skeletal muscle. Proc. Natl. Acad. Sci. USA.

[56] Chen A., Zorrilla E., Smith S., Rousso D., Levy C., Vaughan J., Donaldson C., Roberts A., Lee K.-F., Vale W. (2006). Urocortin 2-Deficient Mice Exhibit Gender-Specific Alterations in Circadian Hypothalamus–Pituitary–Adrenal Axis and Depressive-Like Behavior. J. Neurosci..

[57] Li C., Chen P., Vaughan J., Lee K.-F., Vale W. (2007). Urocortin 3 regulates glucose-stimulated insulin secretion and energy homeostasis. Proc. Natl. Acad. Sci. USA.

[58] Neufeld-Cohen A., Evans A.K., Getselter D., Spyroglou A., Hill A., Gil S., Tsoory M., Beuschlein F., Lowry C.A., Vale W. (2009). Urocortin-1 and -2 double-deficient mice show robust anxiolytic phenotype and modified serotonergic activity in anxiety circuits. Mol. Psychiatry.

[59] Smith G.W., Aubry J.-M., Dellu F., Contarino A., Bilezikjian L.M., Gold L.H., Chen R., Marchuk Y., Hauser C., A Bentley C. (1998). Corticotropin releasing factor receptor 1-deficient mice display decreased anxiety, impaired stress response, and aberrant neuroendocrine development. Neuron.

[60] Bale T.L., Contarino A., Smith G.W., Chan R., Gold L.H., Sawchenko P.E., Koob G.F., Vale W.W., Lee K.-F. (2000). Mice deficient for corticotropin-releasing hormone receptor-2 display anxiety-like behaviour and are hypersensitive to stress. Nat. Genet..

[61] Coste S.C., Kesterson R.A., Heldwein K.A., Stevens S.L., Heard A.D., Hollis J.H., Murray S.E., Hill J.K., Pantely G.A., Hohimer A.R. (2000). Abnormal adaptations to stress and impaired cardiovascular function in mice lacking corticotropin-releasing hormone receptor-2. Nat. Genet..

[62] Bale T.L., Picetti R., Contarino A., Koob G.F., Vale W., Lee K.-F. (2002). Mice Deficient for Both Corticotropin-Releasing Factor Receptor 1 (CRFR1) and CRFR2 Have an Impaired Stress Response and Display Sexually Dichotomous Anxiety-Like Behavior. J. Neurosci..

[63] Sorge R., Martin L.J., Isbester K.A., Sotocinal S.G., Rosen S., Tuttle A.H., Wieskopf J.S., Acland E., Dokova A., Kadoura B. (2014). Olfactory exposure to males, including men, causes stress and related analgesia in rodents. Nat. Methods.

[64] Kim H.J., Prasad V., Hyung S.-W., Lee Z.H., Lee S.-W., Bhargava A., Pearce D., Lee Y., Kim H.-H. (2012). Plasma membrane calcium ATPase regulates bone mass by fine-tuning osteoclast differentiation and survival. J. Cell Boil..

[65] Okunade G.W., Miller M.L., Pyne G.J., Sutliff R.L., O’Connor K.T., Neumann J.C., Andringa A., Miller D.A., Prasad V., Doetschman T. (2004). Targeted Ablation of Plasma Membrane Ca2+-ATPase (PMCA) 1 and 4 Indicates a Major Housekeeping Function for PMCA1 and a Critical Role in Hyperactivated Sperm Motility and Male Fertility for PMCA4. J. Boil. Chem..

[66] Street V.A., McKee-Johnson J.W., Fonseca R.C., Tempel B.L., Noben-Trauth K. (1998). Mutations in a plasma membrane Ca2+-ATPase gene cause deafness in deafwaddler mice. Nat. Genet..

[67] Kozel P.J., Friedman R.A., Erway L.C., Yamoah E.N., Liu L.H., Riddle T., Duffy J.J., Doetschman T., Miller M.L., Cardell E.L. (1998). Balance and Hearing Deficits in Mice with a Null Mutation in the Gene Encoding Plasma Membrane Ca2+-ATPase Isoform 2. J. Boil. Chem..

[68] Hasdemir B., Mahajan S., Bunnett N.W., Liao M., Bhargava A. (2012). Endothelin-converting enzyme-1 actions determine differential trafficking and signaling of corticotropin-releasing factor receptor 1 at high agonist concentrations. Mol. Endocrinol..

[69] Hasdemir B., Mahajan S., Oses-Prieto J., Chand S., Woolley M., Burlingame A., Grammatopoulos D.K., Bhargava A. (2017). Actin cytoskeleton-dependent regulation of corticotropin-releasing factor receptor heteromers. Mol. Biol. Cell.

[70] Bale T.L., Vale W.W. (2004). CRF and CRF receptors: Role in stress responsivity and other behaviors. Annu. Rev. Pharmacol. Toxicol..

[71] Im E., Rhee S.H., Park Y.S., Fiocchi C., Taché Y., Pothoulakis C. (2010). Corticotropin-releasing hormone family of peptides regulates intestinal angiogenesis. YGAST.

[72] Kormos V., Gaszner B. (2013). Role of neuropeptides in anxiety, stress, and depression: From animals to humans. Neuropeptides.

[73] Dautzenberg F.M., Hauger R.L. (2002). The CRF peptide family and their receptors: Yet more partners discovered. Trends Pharmacol. Sci..

[74] Kozicz T., Vigh S., Arimura A. (1997). Axon terminals containing PACAP- and VIP-immunoreactivity form synapses with CRF-immunoreactive neurons in the dorsolateral division of the bed nucleus of the stria terminalis in the rat. Brain Res..

[75] Crestani C.C., Alves F.H., Gomes F., Resstel L.B., Corrêa F., Herman J.P. (2013). Mechanisms in the Bed Nucleus of the Stria Terminalis Involved in Control of Autonomic and Neuroendocrine Functions: A Review. Curr. Neuropharmacol..

[76] Janitzky K., Peine A., Krober A., Yanagawa Y., Schwegler H., Roskoden T. (2014). Increased CRF mRNA expression in the sexually dimorphic BNST of male but not female GAD67 mice and TMT predator odor stress effects upon spatial memory retrieval. Behav. Brain Res..

[77] Sterrenburg L., Gaszner B., Boerrigter J., Santbergen L., Bramini M., Roubos E.W., Peeters B.W., Kozicz T. (2011). Sex-dependent and differential responses to acute restraint stress of corticotropin-releasing factor-producing neurons in the rat paraventricular nucleus, central amygdala, and bed nucleus of the stria terminalis. J. Neurosci. Res..

[78] Sterrenburg L., Gaszner B., Boerrigter J., Santbergen L., Bramini M., Elliott E., Chen A., Peeters B.W., Roubos E.W., Kozicz T. (2011). Chronic stress induces sex-specific alterations in methylation and expression of corticotropin-releasing factor gene in the rat. PLoS ONE.

[79] Gaszner B., Jensen K.O., Farkas J., Reglodi D., Csernus V., Roubos E.W., Kozicz T. (2009). Effects of maternal separation on dynamics of urocortin 1 and brain-derived neurotrophic factor in the rat non-preganglionic Edinger-Westphal nucleus. Int. J. Dev. Neurosci..

[80] Chang J., Hoy J.J., Idumalla P.S., Clifton M.S., Pecoraro N.C., Bhargava A. (2007). Urocortin 2 expression in the rat gastrointestinal tract under basal conditions and in chemical colitis. Peptides.

[81] Hsu S.Y., Hsueh A.J. (2001). Human stresscopin and stresscopin-related peptide are selective ligands for the type 2 corticotropin-releasing hormone receptor. Nat. Med..

[82] Reyes T.M., Lewis K., Perrin M.H., Kunitake K.S., Vaughan J., Arias C.A., HogenEsch J.B., Gulyás J., Rivier J., Vale W. (2001). Urocortin II: A member of the corticotropin-releasing factor (CRF) neuropeptide family that is selectively bound by type 2 CRF receptors. Proc. Natl. Acad. Sci. USA.

[83] Giamouridis D., Gao M.H., Lai N.C., Tan Z., Kim Y.C., Guo T., Miyanohara A., Blankesteijn M.W., Biessen E.A.L., Hammond H.K. (2019). Urocortin 3 Gene Transfer Increases Function of the Failing Murine Heart. Hum. Gene Ther..

[84] Lewis K., Li C., Perrin M.H., Blount A., Kunitake K., Donaldson C., Vaughan J., Reyes T.M., Gulyás J., Fischer W. (2001). Identification of urocortin III, an additional member of the corticotropin-releasing factor (CRF) family with high affinity for the CRF2 receptor. Proc. Natl. Acad. Sci. USA.

[85] Mahajan S., Liao M., Barkan P., Takahashi K., Bhargava A. (2014). Urocortin 3 expression at baseline and during inflammation in the colon: Corticotropin releasing factor receptors cross-talk. Peptides.

[86] Adão R., Mendes-Ferreira P., Santos-Ribeiro D., Maia-Rocha C., Pimentel L., Pinto C., Mulvaney E.P., Reid H.M., Kinsella B.T., Potus F. (2018). Urocortin-2 improves right ventricular function and attenuates pulmonary arterial hypertension. Cardiovasc. Res..

[87] Chatzaki E., Anton P.A., Million M., Lambropoulou M., Constantinidis T., Kolios G., Taché Y., Grigoriadis D.E. (2013). Corticotropin-releasing factor receptor subtype 2 in human colonic mucosa: Down-regulation in ulcerative colitis. World J. Gastroenterol..

[88] Davidson S.M., Rybka A.E., Townsend P.A. (2009). The powerful cardioprotective effects of urocortin and the corticotropin releasing hormone (CRH) family. Biochem. Pharmacol..

[89] Donaldson C.J., Sutton S.W., Perrin M.H., Corrigan A.Z., Lewis K.A., Rivier J.E., Vaughan J.M., Vale W.W. (1996). Cloning and characterization of human urocortin. Endocrinology.

[90] Florio P., Vale W., Petraglia F. (2004). Urocortins in human reproduction. Peptides.

[91] Giamouridis D., Gao M.H., Lai N.C., Tan Z., Kim Y.C., Guo T., Miyanohara A., Blankesteijn W.M., Biessen E., Hammond H.K. (2018). Effects of Urocortin 2 Versus Urocortin 3 Gene Transfer on Left Ventricular Function and Glucose Disposal. JACC Basic Transl. Sci..

[92] Hasdemir B., Mhaske P.V., Paruthiyil S., Garnett E.A., Heyman M.B., Matloubian M., Bhargava A. (2016). Sex-and corticotropin-releasing factor receptor 2- dependent actions of urocortin 1 during inflammation. Am. J. Physiol. Integr. Comp. Physiol..

[93] Kubat E., Mahajan S., Liao M., Ackerman L., Ohara P.T., Grady E.F., Bhargava A. (2013). Corticotropin-releasing factor receptor 2 mediates sex-specific cellular stress responses. Mol. Med..

[94] Florio P., Torricelli M., Galleri L., De Falco G., Leucci E., Calonaci G., Picciolini E., Ambrosini G., Linton E.A., Petraglia F. (2005). High Fetal Urocortin Levels at Term and Preterm Labor. J. Clin. Endocrinol. Metab..

[95] Torricelli M., Ignacchiti E., Giovannelli A., Merola A., Scarpetti E., Calonaci G., Picciolini E., Florio P., Reis F.M., Linton E.A. (2006). Maternal plasma corticotrophin-releasing factor and urocortin levels in post-term pregnancies. Eur. J. Endocrinol..

[96] Vitale S.G., Laganà A.S., Rapisarda A.M.C., Scarale M.G., Corrado F., Cignini P., Butticè S., Rossetti D. (2016). Role of urocortin in pregnancy: An update and future perspectives. World J. Clin. Cases.

[97] Chen R., Lewis K.A., Perrin M.H., Vale W.W. (1993). Expression cloning of a human corticotropin-releasing-factor receptor. Proc. Natl. Acad. Sci. USA.

[98] Perrin M., Donaldson C., Chen R., Blount A., Berggren T., Bilezikjian L., Sawchenko P., Vale W. (1995). Identification of a second corticotropin-releasing factor receptor gene and characterization of a cDNA expressed in heart. Proc. Natl. Acad. Sci. USA.

[99] Parham K.L., Zervou S., Karteris E., Catalano R.D., Old R.W., Hillhouse E. (2004). Promoter Analysis of Human Corticotropin-Releasing Factor (CRF) Type 1 Receptor and Regulation by CRF and Urocortin. Endocrinology.

[100] Catalano R.D., Kyriakou T., Chen J., Easton A., Hillhouse E.W. (2003). Regulation of Corticotropin-Releasing Hormone Type 2 Receptors by Multiple Promoters and Alternative Splicing: Identification of Multiple Splice Variants. Mol. Endocrinol..

[101] Kostich W.A., Chen A., Sperle K., Largent B.L. (1998). Molecular Identification and Analysis of a Novel Human Corticotropin-Releasing Factor (CRF) Receptor: The CRF2γ Receptor. Mol. Endocrinol..

[102] Lovenberg T.W., Chalmers D.T., Liu C., De Souza E.B. (1995). CRF2 alpha and CRF2 beta receptor mRNAs are differentially distributed between the rat central nervous system and peripheral tissues. Endocrinology.

[103] Weiser M.J., Goel N., Sandau U.S., Bale T.L., Handa R.J. (2008). Androgen regulation of corticotropin-releasing hormone receptor 2 (CRHR2) mRNA expression and receptor binding in the rat brain. Exp. Neurol..

[104] Pal K., Swaminathan K., Xu H.E., Pioszak A.A. (2010). Structural basis for hormone recognition by the Human CRFR2{alpha} G protein-coupled receptor. J. Biol. Chem..

[105] Rosinger Z.J., Jacobskind J.S., De Guzman R.M., Justice N.J., Zuloaga D.G. (2019). A sexually dimorphic distribution of corticotropin-releasing factor receptor 1 in the paraventricular hypothalamus. Neuroscience.

[106] Weathington J.M., Hamki A., Cooke B.M. (2014). Sex- and region-specific pubertal maturation of the corticotropin-releasing factor receptor system in the rat. J. Comp. Neurol..

[107] Lim M.M., Nair H.P., Young L.J. (2005). Species and sex differences in brain distribution of corticotropin-releasing factor receptor subtypes 1 and 2 in monogamous and promiscuous vole species. J. Comp. Neurol..

[108] Grace C.R.R., Perrin M.H., Cantle J.P., Vale W.W., Rivier J.E., Riek R. (2007). Common and Divergent Structural Features of a Series of Corticotropin Releasing Factor-Related Peptides. J. Am. Chem. Soc..

[109] Gutknecht E., Vauquelin G., Dautzenberg F.M. (2010). Corticotropin-releasing factor receptors induce calcium mobilization through cross-talk with Gq-coupled receptors. Eur. J. Pharmacol..

[110] Hillhouse E., Grammatopoulos D. (2006). The Molecular Mechanisms Underlying the Regulation of the Biological Activity of Corticotropin-Releasing Hormone Receptors: Implications for Physiology and Pathophysiology. Endocr. Rev..

[111] Oakley R.H., Olivares-Reyes J.A., Hudson C.C., Flores-Vega F., Dautzenberg F.M., Hauger R.L. (2007). Carboxyl-terminal and intracellular loop sites for CRF1 receptor phosphorylation and beta-arrestin-2 recruitment: A mechanism regulating stress and anxiety responses. Am. J. Physiol. Regul. Integr. Comp. Physiol..

[112] Alken M., Rutz C., Köchl R., Donalies U., Oueslati M., Furkert J., Wietfeld D., Hermosilla R., Scholz A., Beyermann M. (2005). The signal peptide of the rat corticotropin-releasing factor receptor 1 promotes receptor expression but is not essential for establishing a functional receptor. Biochem. J..

[113] Rutz C., Renner A., Alken M., Schulz K., Beyermann M., Wiesner B., Rosenthal W., Schülein R. (2006). The Corticotropin-releasing Factor Receptor Type 2a Contains an N-terminal Pseudo Signal Peptide. J. Boil. Chem..

[114] Schulein A.G.A.C.R.R. (2017). Functional Significance of the Signal Peptides of Corticotropin-Releasing Factor Receptors. Curr. Mol. Pharmacol..

[115] Schulz K., Rutz C., Westendorf C., Ridelis I., Vogelbein S., Furkert J., Schmidt A., Wiesner B., Schülein R. (2010). The Pseudo Signal Peptide of the Corticotropin-releasing Factor Receptor Type 2a Decreases Receptor Expression and Prevents Gi-mediated Inhibition of Adenylyl Cyclase Activity. J. Boil. Chem..

[116] Teichmann A., Rutz C., Kreuchwig A., Krause G., Wiesner B., Schülein R. (2012). The Pseudo Signal Peptide of the Corticotropin-releasing Factor Receptor Type 2A Prevents Receptor Oligomerization. J. Boil. Chem..

[117] Resh M.D. (1999). Fatty acylation of proteins: New insights into membrane targeting of myristoylated and palmitoylated proteins. Biochim. Biophys. Acta (BBA) Bioenerg..

[118] Binda A.V., Kabbani N., Lin R., Levenson R. (2002). D2 and D3 Dopamine Receptor Cell Surface Localization Mediated by Interaction with Protein 4.1N. Mol. Pharmacol..

[119] Cornea-Hébert V., Watkins K.C., Roth B.L., Kroeze W.K., Gaudreau P., Leclerc N., Descarries L. (2002). Similar ultrastructural distribution of the 5-HT2A serotonin receptor and microtubule-associated protein MAP1A in cortical dendrites of adult rat. Neuroscience.

[120] Kim O.-J., Ariano M.A., Lazzarini R.A., Levine M.S., Sibley D.R. (2002). Neurofilament-M Interacts with the D1 Dopamine Receptor to Regulate Cell Surface Expression and Desensitization. J. Neurosci..

[121] Inda C., Armando N.G., Claro P.A.D.S., Silberstein S. (2017). Endocrinology and the brain: Corticotropin-releasing hormone signaling. Endocr. Connect..

[122] Mikhailova M.V., Mayeux P.R., Jurkevich A., Kuenzel W.J., Madison F., Periasamy A., Chen Y., Cornett L.E. (2007). Heterooligomerization between vasotocin and corticotropin-releasing hormone (CRH) receptors augments CRH-stimulated 3′,5′-cyclic adenosine monophosphate production. Mol. Endocrinol..

[123] Murat B., Devost D., Andrés M., Mion J., Boulay V., Corbani M., Zingg H.H., Guillon G. (2012). V1b and CRHR1 Receptor Heterodimerization Mediates Synergistic Biological Actions of Vasopressin and CRH. Mol. Endocrinol..

[124] Magalhaes A.C., Holmes K.D., Dale L.B., Comps-Agrar L., Lee D., Yadav P.N., Drysdale L., O Poulter M., Roth B.L., Pin J.-P. (2010). CRF receptor 1 regulates anxiety behavior via sensitization of 5-HT2 receptor signaling. Nat. Neurosci..

[125] Bangasser D.A., Curtis A., Reyes B.A., Bethea T.T., Parastatidis I., Ischiropoulos H., Van Bockstaele E.J., Valentino R.J. (2010). Sex differences in corticotropin-releasing factor receptor signaling and trafficking: Potential role in female vulnerability to stress-related psychopathology. Mol. Psychiatry.

[126] Rademaker M.T., Richards A.M. (2017). Urocortins: Actions in health and heart failure. Clin. Chim. Acta.

[127] Walczewska J., Dzieza-Grudnik A., Siga O., Grodzicki T. (2014). The role of urocortins in the cardiovascular system. J. Physiol. Pharmacol. Off. J. Pol. Physiol. Soc..

[128] Yang L.-Z., Chen Y. (2019). Research on the Effects of the Chronic Treatment with Different Doses of Urocortin 2 in Heart Failure Rats. Dose-Response.

[129] Yang L.-Z., Tovote P., Rayner M., Kockskämper J., Pieske B., Spiess J. (2010). Corticotropin-releasing factor receptors and urocortins, links between the brain and the heart. Eur. J. Pharmacol..

[130] Cserepes B., Jancsó G., Gasz B., Rácz B., Ferencz A., Benkó L., Borsiczky B., Kürthy M., Ferencz S., Lantos J. (2007). Cardioprotective Action of Urocortin in Early Pre- and Postconditioning. Ann. N. Y. Acad. Sci..

[131] Díaz I., Calderón-Sánchez E.M., Del Toro R., Avila-Medina J., Pedro E.S.D.R.-D., Domínguez-Rodríguez A., Rosado J.A., Hmadcha A., Ordóñez A., Smani T. (2017). miR-125a, miR-139 and miR-324 contribute to Urocortin protection against myocardial ischemia-reperfusion injury. Sci. Rep..

[132] Yang L.-Z., Kockskämper J., Heinzel F.R., Hauber M., Walther S., Spiess J., Pieske B. (2006). Urocortin II enhances contractility in rabbit ventricular myocytes via CRF(2) receptor-mediated stimulation of protein kinase A. Cardiovasc. Res..

[133] Domínguez-Rodríguez A., Mayoral-Gonzalez I., Avila-Medina J., Pedro E.S.D.R.-D., Calderón-Sánchez E., Díaz I., Hmadcha A., Castellano A., Rosado J.A., Benitah J.-P. (2018). Urocortin-2 Prevents Dysregulation of Ca2+ Homeostasis and Improves Early Cardiac Remodeling After Ischemia and Reperfusion. Front. Physiol..

[134] Albrektsen G., Heuch I., Løchen M.-L., Thelle D.S., Wilsgaard T., Njølstad I., Bønaa K.H. (2016). Lifelong Gender Gap in Risk of Incident Myocardial Infarction: The Tromsø Study. JAMA Intern. Med..

[135] Gallucci W.T., Baum A., Laue L., Rabin D.S., Chrousos G.P., Gold P.W., Kling M.A. (1993). Sex differences in sensitivity of the hypothalamic-pituitary-adrenal axis. Health Psychol..

[136] Aston-Jones G., Rajkowski J., Kubiak P., Valentino R.J., Shipley M.T. (1996). Chapter 23 Role of the Locus Coeruleus in Emotional Activation.

[137] Page M.E., Berridge C.W., Foote S.L., Valentino R.J. (1993). Corticotropin-releasing factor in the locus coeruleus mediates EEG activation associated with hypotensive stress. Neurosci. Lett..

[138] Van Bockstaele E.J., Reyes B.A.S., Valentino R.J. (2009). The locus coeruleus: A key nucleus where stress and opioids intersect to mediate vulnerability to opiate abuse. Brain Res..

[139] García R.M.F., Pinos H., Collado P., Pásaro E., Fernández R., Segovia S., Guillamon A. (2005). The expression of brain sexual dimorphism in artificial selection of rat strains. Brain Res..

[140] Garcia-Falgueras A., Pinos H., Fernandez R., Collado P., Pasaro E., Segovia S., Guillamon A. (2006). Sexual dimorphism in hybrids rats. Brain Res..

[141] Bangasser D.A., Zhang X., Garachh V., Hanhauser E., Valentino R.J. (2011). Sexual dimorphism in locus coeruleus dendritic morphology: A structural basis for sex differences in emotional arousal. Physiol. Behav..

[142] Wiersielis K.R., Wicks B., Simko H., Cohen S.R., Khantsis S., Baksh N., Waxler D.E., Bangasser D. (2016). Sex differences in corticotropin releasing factor-evoked behavior and activated networks. Psychoneuroendocrinology.

[143] Toth M., Flandreau E.I., DesLauriers J., Geyer M.A., Mansuy I.M., Merlo-Pich E., Risbrough V.B. (2015). Overexpression of Forebrain CRH During Early Life Increases Trauma Susceptibility in Adulthood. Neuropsychopharmacology.

[144] Haeger P., Andres M.E., Forray M.I., Daza C., Araneda S., Gysling K. (2006). Estrogen receptors alpha and beta differentially regulate the transcriptional activity of the Urocortin gene. J. Neurosci..

[145] Fonareva I., Spangler E., Cannella N., Sabino V., Cottone P., Ciccocioppo R., Zorrilla E.P., Ryabinin A.E. (2009). Increased perioculomotor urocortin 1 immunoreactivity in genetically selected alcohol preferring rats. Alcohol. Clin. Exp. Res..

[146] Fatima A., Haroon M., Wolf G., Engelmann M., Spina M. (2007). Reduced urocortin 1 immunoreactivity in the non-preganglionic Edinger–Westphal nucleus during late pregnancy in rats. Regul. Pept..

[147] Kozicz T., Tilburg-Ouwens D., Faludi G., Palkovits M., Roubos E. (2008). Gender-related urocortin 1 and brain-derived neurotrophic factor expression in the adult human midbrain of suicide victims with major depression. Neuroscience.

[148] Wolf E., Mitchell K.S., Logue M.W., Baldwin C.T., Reardon A.F., Humphries D.E., Miller M. (2013). Corticotropin Releasing Hormone Receptor 2 (Crhr-2) Gene Is Associated with Decreased Risk and Severity of Posttraumatic Stress Disorder in Women. Depression Anxiety.

[149] Bangasser D., Valentino R.J. (2014). Sex differences in stress-related psychiatric disorders: Neurobiological perspectives. Front. Neuroendocr..

[150] Liu Z., Zhu F., Wang G., Xiao Z., Wang H., Tang J., Wang X., Qiu D., Liu W., Cao Z. (2006). Association of corticotropin-releasing hormone receptor1 gene SNP and haplotype with major depression. Neurosci. Lett..

[151] Engineer N., Darwin L., Nishigandh D., Ngianga-Bakwin K., Smith S.C., Grammatopoulos D. (2013). Association of glucocorticoid and type 1 corticotropin-releasing hormone receptors gene variants and risk for depression during pregnancy and post-partum. J. Psychiatr. Res..

[152] Orand A., Naliboff B., Gadd M., Shih W., Ju T., Presson A.P., Mayer E.A., Chang L. (2016). Corticotropin-releasing hormone receptor 1 (CRH-R1) polymorphisms are associated with irritable bowel syndrome and acoustic startle response. Psychoneuroendocrinology.

[153] Bradley R.G., Binder E.B., Epstein M.P., Tang Y., Nair H.P., Liu W., Gillespie C., Berg T., Evces M., Newport D.J. (2008). Influence of Child Abuse on Adult Depression. Arch. Gen. Psychiatry.

[154] Polanczyk G.V., Caspi A., Williams B., Price T., Danese A., Sugden K., Uher R., Poulton R., Moffitt T.E. (2009). Protective effect of CRHR1 gene variants on the development of adult depression following childhood maltreatment: Replication and extension. Arch. Gen. Psychiatry.

[155] Heim C., Bradley B., Mletzko T.C., Deveau T.C., Musselman D.L., Nemeroff C.B., Ressler K.J., Binder E.B. (2009). Effect of Childhood Trauma on Adult Depression and Neuroendocrine Function: Sex-Specific Moderation by CRH Receptor 1 Gene. Front. Behav. Neurosci..

[156] Mulak A., Taché Y., Larauche M. (2014). Sex hormones in the modulation of irritable bowel syndrome. World J. Gastroenterol..

[157] Million M., Maillot C., Saunders P., Rivier J., Vale W., Taché Y. (2002). Human urocortin II, a new CRF-related peptide, displays selective CRF2-mediated action on gastric transit in rats. Am. J. Physiol. Liver Physiol..

[158] Taché Y., Martinez V., Wang L., Million M. (2004). CRF1 receptor signaling pathways are involved in stress-related alterations of colonic function and viscerosensitivity: Implications for irritable bowel syndrome. Br. J. Pharmacol..

[159] Hagiwara S.I., Kaushal E., Paruthiyil S., Pasricha P.J., Hasdemir B., Bhargava A. (2018). Gastric corticotropin-releasing factor influences mast cell infiltration in a rat model of functional dyspepsia. PLoS ONE.

[160] Hagiwara S.-I., Hasdemir B., Heyman M.B., Chang L., Bhargava A. (2019). Plasma Corticotropin-Releasing Factor Receptors and B7-2+ Extracellular Vesicles in Blood Correlate with Irritable Bowel Syndrome Disease Severity. Cells.

[161] Wallon C., Yang P.-C., Keita Åsa, Ericson A.-C., McKay D.M., Sherman P.M., Perdue M.H., Söderholm J.D. (2007). Corticotropin-releasing hormone (CRH) regulates macromolecular permeability via mast cells in normal human colonic biopsies in vitro. Gut.

[162] Tsatsanis C., Androulidaki A., Alissafi T., Charalampopoulos I., Dermitzaki E., Roger T., Gravanis A., Margioris A.N. (2006). Corticotropin-releasing factor and the urocortins induce the expression of TLR4 in macrophages via activation of the transcription factors PU.1 and AP-1. J. Immunol..

[163] Tsatsanis C., Androulidaki A., Dermitzaki E., Gravanis A., Margioris A.N. (2006). Corticotropin releasing factor receptor 1 (CRF1) and CRF2 agonists exert an anti-inflammatory effect during the early phase of inflammation suppressing LPS-induced TNF-α release from macrophages via induction of COX-2 and PGE2. J. Cell. Physiol..

[164] Chatzaki E. (2003). Urocortin in Human Gastric Mucosa: Relationship to Inflammatory Activity. J. Clin. Endocrinol. Metab..

[165] Muramatsu Y., Fukushima K., Iino K., Totsune K., Takahashi K., Suzuki T., Hirasawa G., Takeyama J., Ito M., Nose M. (2000). Urocortin and corticotropin-releasing factor receptor expression in the human colonic mucosa. Peptides.

[166] Saruta M., Takahashi K., Suzuki T., Fukuda T., Torii A., Sasano H. (2005). Urocortin 3/stresscopin in human colon: Possible modulators of gastrointestinal function during stressful conditions. Peptides.

[167] Yuan J., Hasdemir B., Tan T., Chheda C., Rivier J., Pandol S.J., Bhargava A. (2019). Protective effects of urocortin 2 against caerulein-induced acute pancreatitis. PLoS ONE.

[168] Kautzky-Willers A., Harreiter J., Pacini G. (2016). Sex and Gender Differences in Risk, Pathophysiology and Complications of Type 2 Diabetes Mellitus. Endocr. Rev..

[169] Bergmann N.C., Gyntelberg F., Faber J. (2014). The appraisal of chronic stress and the development of the metabolic syndrome: A systematic review of prospective cohort studies. Endocr. Connect..

[170] Pouwer F., Kupper N., Adriaanse M.C. (2010). Does emotional stress cause type 2 diabetes mellitus? A review from the European Depression in Diabetes (EDID) Research Consortium. Discov. Med..

[171] Mahajan A., Go M.J., Zhang W., Below J., Gaulton K.J., Ferreira T., Horikoshi M., Johnson A.D., Ng M.C.Y., DIAbetes Genetics Replication And Meta-analysis (DIAGRAM) Consortium (2014). Genome-wide trans-ancestry meta-analysis provides insight into the genetic architecture of type 2 diabetes susceptibility. Nat. Genet..

[172] Kautzky-Willers A., Harreiter J. (2017). Sex and gender differences in therapy of type 2 diabetes. Diabetes Res. Clin. Pr..

[173] Ingvorsen C., Karp N., Lelliott C.J. (2017). The role of sex and body weight on the metabolic effects of high-fat diet in C57BL/6N mice. Nutr. Diabetes.

[174] Viau V., Bingham B., Davis J., Lee P., Wong M. (2005). Gender and Puberty Interact on the Stress-Induced Activation of Parvocellular Neurosecretory Neurons and Corticotropin-Releasing Hormone Messenger Ribonucleic Acid Expression in the Rat. Endocrinology.

[175] Figueiredo H.F., Ulrich-Lai Y., Choi D.C., Herman J.P. (2007). Estrogen potentiates adrenocortical responses to stress in female rats. Am. J. Physiol. Metab..

[176] Bao A.-M., Swaab D.F. (2007). Gender Difference in Age-Related Number of Corticotropin-Releasing Hormone-Expressing Neurons in the Human Hypothalamic Paraventricular Nucleus and the Role of Sex Hormones. Neuroendocrinology.

[177] Uchida K., Otsuka H., Morishita M., Tsukahara S., Sato T., Sakimura K., Itoi K. (2019). Female-biased sexual dimorphism of corticotropin-releasing factor neurons in the bed nucleus of the stria terminalis. Biol. Sex. Differ..

[178] Perkins A., Linton E.A., Eben F., Simpson J., Wolfe C.D.A., Redman C.W.G. (1995). Corticotrophin-releasing hormone and corticotrophin- releasing hormone binding protein in normal and pre-eclamptic human pregnancies. BJOG: Int. J. Obstet. Gynaecol..

[179] Imperatore A., Rolfo A., Petraglia F., Challis J.R., Caniggia I. (2010). Hypoxia and preeclampsia: Increased expression of urocortin 2 and urocortin 3. Reprod. Sci..

[180] Bamberger C.M., Minas V., Bamberger A.M., Charalampopoulos I., Fragouli Y., Schulte H.M., Makrigiannakis A. (2007). Expression of urocortin in the extravillous human trophoblast at the implantation site. Placenta.

[181] Florio P., Bruni L., De Falco C., Filardi G., Torricelli M., Reis F.M., Galleri L., Voltolini C., Bocchi C., De Leo V. (2008). Evaluation of Endometrial Urocortin Secretion for Prediction of Pregnancy after Intrauterine Insemination. Clin. Chem..

[182] Florio P., Calonaci G., Severi F.M., Torricelli M., Bocchi C., Fiore G., Linton E.A., Petraglia F. (2005). Reduced Maternal Plasma Urocortin Concentrations and Impaired Uterine Artery Blood Flow at Human Mid Pregnancy. J. Soc. Gynecol. Investig..

[183] Florio P., Linton E.A., Torricelli M., Faldini E., Reis F.M., Imperatore A., Calonaci G., Picciolini E., Petraglia F. (2007). Prediction of Preterm Delivery Based on Maternal Plasma Urocortin. J. Clin. Endocrinol. Metab..

[184] Florio P., Torricelli M., De Falco G., Leucci E., Giovannelli A., Gazzolo D., Severi F.M., Bagnoli F., Leoncini L., A Linton E. (2006). High maternal and fetal plasma urocortin levels in pregnancies complicated by hypertension. J. Hypertens..

[185] Mourad M., Haitham A., Mohsen S., Hani M.M. (2016). Value of Urocortin as a Marker for Preterm Labor. J. Gynecol. Women’s Health.

[186] Iavazzo C., Tassis K., Gourgiotis D., Boutsikou M., Baka S., Hassiakos D., Vogiatzi C., Florentin-Arar L., Malamitsi-Puchner A. (2009). Urocortin in Second Trimester Amniotic Fluid: Its Role as Predictor of Preterm Labor. Mediat. Inflamm..

[187] La Marca-Ghaemmaghami P., Dainese S.M., Stalla G., Haller M., Zimmermann R., Ehlert U. (2017). Second-Trimester Amniotic Fluid Corticotropin-Releasing Hormone and Urocortin in Relation to Maternal Stress and Fetal Growth in Human Pregnancy. Stress.

[188] Karaer A., Celik E., Celik O., Simsek O.Y., Ozerol I.H., Yılmaz E., Türkçüoğlu I., Duz S.A. (2013). Amniotic fluid urocortin-1 concentrations for the prediction of preterm delivery. J. Obstet. Gynaecol. Res..

[189] Torricelli M., Novembri R., Bloise E., De Bonis M., Challis J.R., Petraglia F. (2011). Changes in Placental CRH, Urocortins, and CRH-Receptor mRNA Expression Associated with Preterm Delivery and Chorioamnionitis. J. Clin. Endocrinol. Metab..

[190] Kashanian M., Bahasadri S., Ghasemi A., Bathaee S. (2012). Value of serum urocortin concentration in the prediction of preterm birth. J. Obstet. Gynaecol. Res..

[191] Sandman C.A., Glynn L., Schetter C.D., Wadhwa P., Garite T., Chicz-DeMet A., Hobel C. (2006). Elevated maternal cortisol early in pregnancy predicts third trimester levels of placental corticotropin releasing hormone (CRH): Priming the placental clock. Peptides.

[192] Asakura H., Zwain I.H., Yen S.S. (1997). Expression of genes encoding corticotropin-releasing factor (CRF), type 1 CRF receptor, and CRF-binding protein and localization of the gene products in the human ovary. J. Clin. Endocrinol. Metab..

[193] Muramatsu Y., Sugino N., Suzuki T., Totsune K., Takahashi K., Tashiro A., Hongo M., Oki Y., Sasano H. (2001). Urocortin and corticotropin-releasing factor receptor expression in normal cycling human ovaries. J. Clin. Endocrinol. Metab..

[194] Torricelli M., De Falco G., Florio P., Rossi M., Leucci E., Viganó P., Leoncini L., Petraglia F. (2006). Secretory endometrium highly expresses urocortin messenger RNA and peptide: Possible role in the decidualization process. Hum. Reprod..

[195] Florio P., Reis F.M., Torres P.B., Calonaci F., Toti P., Bocchi C., Linton E.A., Petraglia F. (2007). Plasma Urocortin Levels in the Diagnosis of Ovarian Endometriosis. Obstet. Gynecol..

[196] Dunn-Fletcher C., Muglia L.M., Pavlicev M., Wolf G., Sun M.-A., Hu Y.-C., Huffman E., Tumukuntala S., Thiele K., Mukherjee A. (2018). Anthropoid primate-specific retroviral element THE1B controls expression of CRH in placenta and alters gestation length. PLoS Boil..

